# Salmonella enterica Infections Are Disrupted by Two Small Molecules That Accumulate within Phagosomes and Differentially Damage Bacterial Inner Membranes

**DOI:** 10.1128/mbio.01790-22

**Published:** 2022-09-22

**Authors:** Joseph A. Villanueva, Amy L. Crooks, Toni A. Nagy, Joaquin L. J. Quintana, Zachary D. Dalebroux, Corrella S. Detweiler

**Affiliations:** a Department of Molecular, Cellular, and Developmental Biology, University of Colorado Boulder, Boulder, Colorado, USA; b Department of Microbiology and Immunology, University of Oklahoma Health Sciences Center, Oklahoma City, Oklahoma, USA; Princeton University; Fred Hutchinson Cancer Center

**Keywords:** amine substituted, antibacterial, anti-infective, *Galleria mellonella*, inner membrane, intracellular pathogen, intravesicular pathogen, lysosomal trapping, macrophage, *Salmonella enterica*

## Abstract

Gram-negative bacteria have a robust cell envelope that excludes or expels many antimicrobial agents. However, during infection, host soluble innate immune factors permeabilize the bacterial outer membrane. We identified two small molecules that exploit outer membrane damage to access the bacterial cell. In standard microbiological media, neither compound inhibited bacterial growth nor permeabilized bacterial outer membranes. In contrast, at micromolar concentrations, JAV1 and JAV2 enabled the killing of an intracellular human pathogen, Salmonella enterica serovar Typhimurium. *S.* Typhimurium is a Gram-negative bacterium that resides within phagosomes of cells from the monocyte lineage. Under broth conditions that destabilized the lipopolysaccharide layer, JAV2 permeabilized the bacterial inner membrane and was rapidly bactericidal. In contrast, JAV1 activity was more subtle: JAV1 increased membrane fluidity, altered reduction potential, and required more time than JAV2 to disrupt the inner membrane barrier and kill bacteria. Both compounds interacted with glycerophospholipids from Escherichia coli total lipid extract-based liposomes. JAV1 preferentially interacted with cardiolipin and partially relied on cardiolipin production for activity, whereas JAV2 generally interacted with lipids and had modest affinity for phosphatidylglycerol. In mammalian cells, neither compound significantly altered mitochondrial membrane potential at concentrations that killed *S.* Typhimurium. Instead, JAV1 and JAV2 became trapped within acidic compartments, including macrophage phagosomes. Both compounds improved survival of *S.* Typhimurium-infected Galleria mellonella larvae. Together, these data demonstrate that JAV1 and JAV2 disrupt bacterial inner membranes by distinct mechanisms and highlight how small, lipophilic, amine-substituted molecules can exploit host soluble innate immunity to facilitate the killing of intravesicular pathogens.

## INTRODUCTION

The discovery of novel antimicrobial chemical scaffolds and the development of new antimicrobials has slowed in recent decades for a variety of economic, regulatory, and societal reasons (1). New antibiotics are needed for infections caused by Gram-negative bacterial pathogens, because these organisms are especially impermeable to chemicals (2). Gram-negative bacteria are enshrouded by two membranes that together prevent drug entry (3). The outer membrane is a selective barrier with an asymmetric architecture. The outer leaflet of the outer membrane mainly consists of negatively charged lipopolysaccharides (LPS), while the inner leaflet is comprised of glycerophospholipids. The inner membrane is symmetrical and composed of glycerophospholipids, which for *Enterobacteriaceae* primarily include the zwitterionic phosphatidylethanolamines (PE), anionic phosphatidylglycerols (PG), and cardiolipins (CL) (4–6). Between the dual bilayers is a porous peptidoglycan cell wall, which is attached to the outer membrane by lipoproteins. Together, these three layers constitute the major structural elements of the cell envelope.

Salmonella enterica serovar Typhimurium (*S.* Typhimurium) is a Gram-negative enterobacterial pathogen that causes gastroenteritis in healthy humans and systemic intracellular disease in immunocompromised individuals and a range of animals. During systemic infection, *S.* Typhimurium typically resides in phagocytic cells of the monocyte lineage (7). In macrophages, *S.* Typhimurium is contained within a specialized acidified vesicular compartment which is referred to as the Salmonella-containing vacuole (SCV) (8). A major *S.* Typhimurium virulence strategy involves regulating the association of the SCV with host late endosomes and lysosomes (9). When the SCV fuses with lysosomes, the bacteria are exposed to innate immunity killing mechanisms, including a pH of <5.0, nutrient starvation, antimicrobial peptides, reactive oxygen and nitrogen species, acid-activated proteases, lysozyme, and complement (10–12). Within the SCV, host immunity creates an environment that destabilizes and disrupts the bacterial cell envelope and helps clear infection.

We previously screened a drug-like compound library by using the SAFIRE (screen for anti-infectives using fluorescence microscopy for intracellular *Enterobacteriaceae*) platform (13). SAFIRE identifies compounds that prevent *S.* Typhimurium accumulation within macrophages, and a secondary screen revealed those which enabled the killing of intracellular bacteria. This approach identified molecules that are not antibacterials but that work with host innate immunity to kill bacteria and are therefore anti-infectives. These compounds include efflux pump inhibitors, a stimulator of macrophage autophagy, and two compounds that damage bacterial inner membranes and reduce bacterial tissue load in mice (13–17). Here, we describe two additional membrane-active, anti-infective compounds, JAV1 and JAV2, which notably have amine groups that are protonatable near pH 6, suggesting compound sequestration within low-pH vesicles, such as the SCV (18, 19). We tested the effects of these compounds on bacteria, mammalian cells, and in the wax moth, Galleria mellonella, a larvae infection model. Our results suggest that small, lipophilic, amine-substituted molecules that differentially damage bacterial inner membranes can accumulate within SCVs, potentiate innate immunity, and facilitate the death of an intravacuolar pathogen.

## RESULTS

### JAV1 and JAV2 prevent survival of *S.* Typhimurium in macrophages.

**FIG 1:**
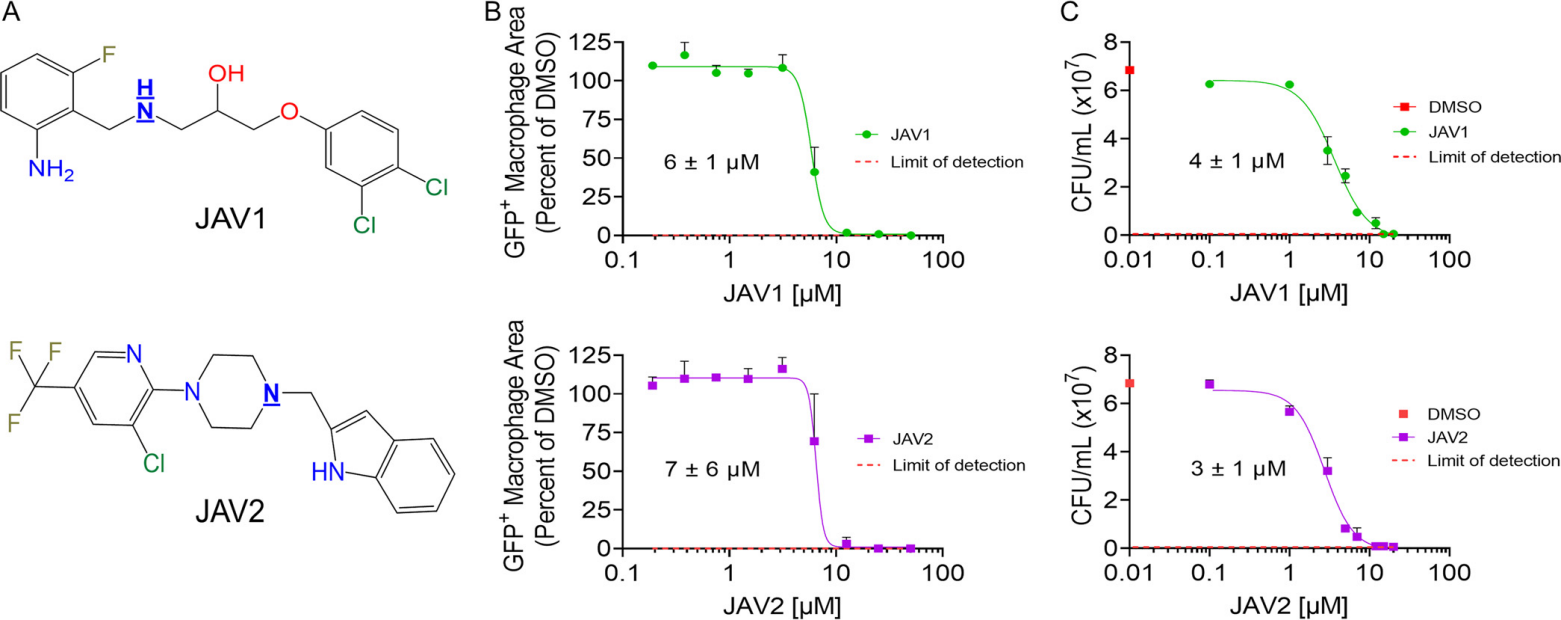
The lipophilic, amine-substituted molecules JAV1 and JAV2 prevent survival of *S.* Typhimurium in macrophages. (A) JAV1 (top) and JAV2 (bottom) chemical structures. The underlined nitrogen atoms are ionizable at pH of <6. (B and C) RAW 264.7 macrophage-like cells were infected with *S.* Typhimurium harboring a chromosomal *sifB*::*gfp* reporter (B) or wild-type *S.* Typhimurium (C). Cells were treated 2 h after infection with DMSO, JAV1, or JAV2 and incubated for 16 h. IC_50_ values are indicated under the curves. Red dashed lines represent the limits of detection. For panel B, cells were analyzed for GFP^+^ macrophage area, defined as the number of GFP^+^ pixels per macrophage divided by the total number of pixels per macrophage, averaged across all macrophages. Means and standard deviations (SD) of two biological replicates performed in technical triplicate are shown. For panel C, cells were lysed and plated for enumeration of CFU. Red squares on the *y* axis represent DMSO-treated samples. Means and standard errors of the means (SEM) of biological triplicates performed in technical duplicate are shown.

From the original SAFIRE screen, we selected JAV1 and JAV2 for examination because they had distinct chemical structures (Fig. 1A) and were potent in preliminary studies (13). To validate their antibacterial activities in macrophages, we repurchased the compounds and performed dose-response experiments using the SAFIRE assay. After 16 h of compound treatment, infected macrophages were monitored for *sifB*::*gfp*-expressing *S.* Typhimurium, mitochondrial membrane potential, and nuclear DNA. The *sifB* promoter is induced within macrophages, such that the *sifB*::*gfp* reporter gene enables detection of intracellular bacteria (13, 20). Green fluorescent protein-positive (GFP^+^) pixels within a macrophage area were quantified, and half-maximal inhibitory concentrations (IC_50_) of 6 to 7 μM were calculated (Fig. 1B). To distinguish whether the compounds interfered with GFP signal or facilitated bacterial killing, macrophages were infected with wild-type bacteria, treated with JAV1 or JAV2, lysed, and plated for CFU counts. Observed reductions in bacterial colonization were at least 100-fold for both methods (see Fig. S1 in the supplemental material), and calculated CFU and SAFIRE IC_50_ values were similar (Fig. 1C). Thus, JAV1 and JAV2 reduced *S.* Typhimurium survival within macrophages at micromolar concentrations.

10.1128/mbio.01790-22.1 FIG S1 JAV1 and JAV2 reduced *S.* Typhimurium accumulation and survival in macrophages by 100-fold. Data from Fig. 1B and C with a log_10_ scale *y*-axis show the reduction in GFP signal (A) and CFU (B). Red dashed lines represent the limits of detection. Download FIG S1, PDF file, 0.3 MB. Copyright © 2022 Villanueva et al. 2022 Villanueva et al. https://creativecommons.org/licenses/by/4.0/ This content is distributed under the terms of the Creative Commons Attribution 4.0 International license.

### Neither JAV1 nor JAV2 significantly permeabilized the outer membrane.

**FIG 2:**
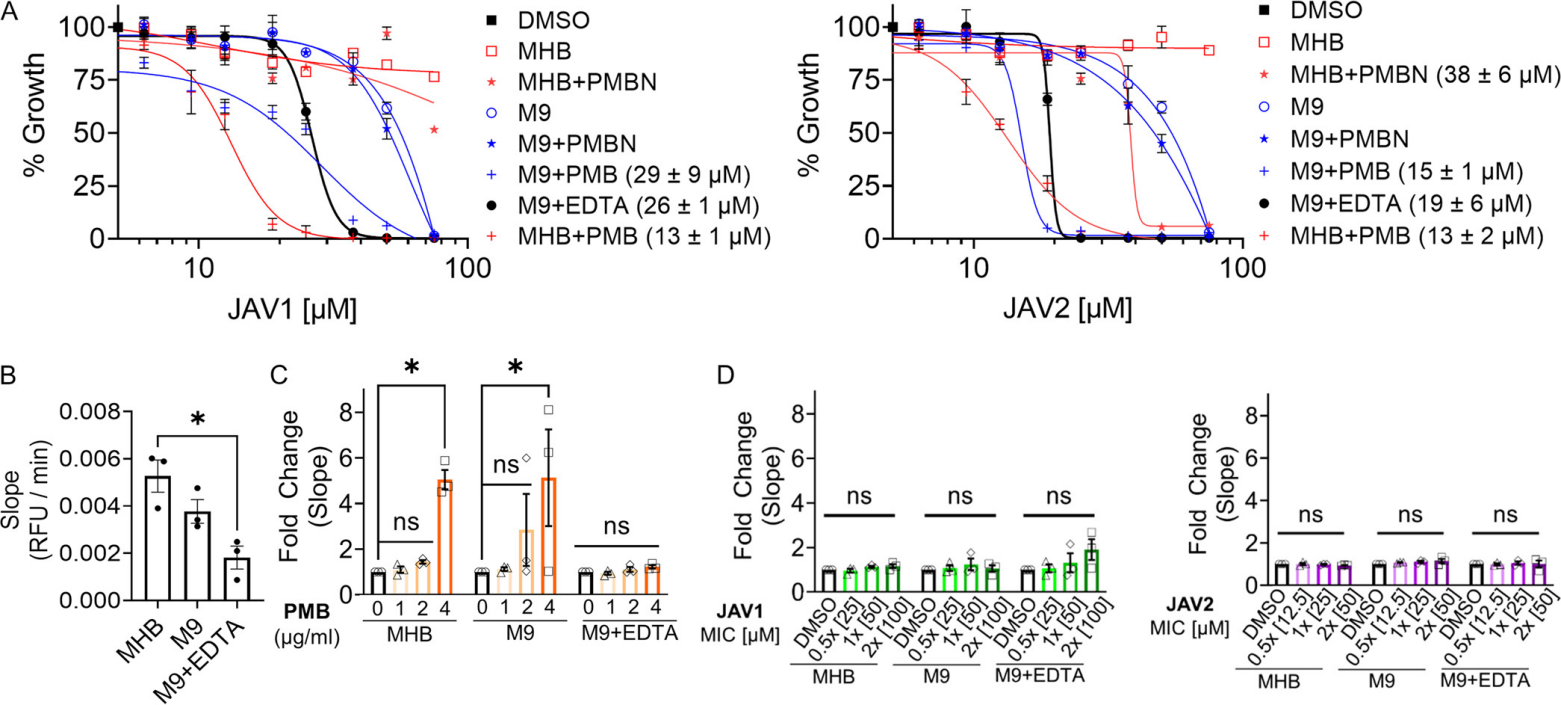
Media that limited nutrients or compromised LPS allowed JAV1 and JAV2 to inhibit *S.* Typhimurium growth in broth, but neither compound permeabilized outer membranes. (A) Growth of *S.* Typhimurium in different media with JAV1 (left) or JAV2 (right). Absorbance (OD_600_) values were normalized to that of DMSO (100%; black square). MHB (red) or M9 (blue) was supplemented as indicated by the keys with polymyxin B (PMB, 1 μg/mL), polymyxin B nonapeptide (PMBN, 30 μg/mL), or EDTA (400 μM, 117 μg/mL). Means and SEM of three biological replicates performed in technical triplicate are shown. IC_50_ values are indicated in the key in parentheses. (B to D) Mid-log-phase *S.* Typhimurium cultures were combined with 100 μM nitrocefin and DMSO (B), PMB (C), or JAV1 (left) or JAV2 (right) (D) in the indicated media and monitored for absorbance (at 486 nm) over 55 min to calculate the slopes (relative fluorescence units [RFU] per minute). Means and SEM of three biological replicates performed in technical triplicate are shown. Data in panelsC and D were normalized to DMSO. For data in panels B to D, * indicates *P < *0.05 (determined by ordinary one-way analysis of variance [ANOVA] and Tukey’s multiple-comparison test) and ns indicates nonsignificant. The overall *P* and *F* values and total degrees of freedom were as follows: 0.0141, 9.4, and 3 (B); <0.50, 3.79, and 2 (C); 0.2, 1.7, and 2 (D), respectively.

JAV1 and JAV2 did not have antibacterial activity in standard, nutrient-rich, cation-adjusted Mueller-Hinton broth (MHB) (Fig. 2A), which was consistent with other compounds identified with the SAFIRE assay (13–15, 21). We also noted that low pH, a characteristic of the phagolysosome, did not potentiate either compound (see Fig. S2). However, we hypothesized that JAV1 and JAV2 could have enabled *S.* Typhimurium killing in macrophages by damaging the outer membrane. Therefore, we determined whether the compounds allows nitrocefin to cross the outer membrane, which results in a color change after cleavage by a periplasmic β-lactamase (22–24). We quantified background nitrocefin access in MHB, M9 minimal medium (M9), and M9 + 400 μM ethylenediaminetetraacetic acid (M9 + EDTA). We found that MHB allowed for the most nitrocefin cleavage of the three conditions (Fig. 2B). In control experiments, treatment of MHB- or M9-grown *S.* Typhimurium with the cationic antimicrobial peptide polymyxin B (PMB; 4 μg/mL) enabled significant nitrocefin cleavage (Fig. 2C), consistent with PMB-induced damage to the LPS layer (25). Chelation of divalent cations by EDTA induced an anticipated resilience to PMB, likely by signaling *S.* Typhimurium to modify LPS lipid A and distal polysaccharides, which increase outer membrane lipid packing and shield negative surface charges, respectively (26–28). In contrast, treatment with JAV1 or JAV2 did not affect nitrocefin access at twice the MIC required to prevent 99% of *S.* Typhimurium growth (2× MIC_99_) (Fig. 2D). These data indicated that neither compound significantly disturbed an outer membrane harboring either standard or PMB-resistant LPS.

10.1128/mbio.01790-22.2 FIG S2 Low pH did not potentiate growth inhibition by JAV1 or JAV2. Growth of *S.* Typhimurium in MHB (A) or M9 (B) with dose titrations of ampicillin (Amp), JAV1 (green), or JAV2 (purple). Amp was included as a control for pH; *S.* Typhimurium is less sensitive to Amp at low versus neutral pH (P. A. Anton, J. A. Kemp, T. Butler, and M. R. Jacobs, Antimicrob Agents Chemother 22:312–315, 1982, https://doi.org/10.1128/AAC.22.2.312; R. Laub, Y.-J. Schneider, and A. Trouet, J Gen Microbiol 135:1407–61406, https://doi.org/10.1099/00221287-135-6-1407). Absorbance (OD_600_) values were normalized to that of the DMSO control (100%). Means and SEM of three biological replicates performed in technical triplicate are shown. Media were adjusted to pH 5.0 with acetic acid (0.2 M) and sodium acetate buffer. In M9 + EDTA at pH 5.0, *S.* Typhimurium did not reach an OD_600_ of 0.4 within 6 h, and therefore this medium was not included. Download FIG S2, PDF file, 0.2 MB. Copyright © 2022 Villanueva et al. 2022 Villanueva et al. https://creativecommons.org/licenses/by/4.0/ This content is distributed under the terms of the Creative Commons Attribution 4.0 International license.

### Limitation of nutrients or destabilization of LPS enabled JAV1 and JAV2 to inhibit *S.* Typhimurium growth.

Previous SAFIRE-identified compounds inhibit bacterial growth under conditions that mimic aspects of the SCV microenvironment, such as nutrient limitation (15, 21). Similarly, in M9 both JAV1 and JAV2 inhibited growth (Fig. 2A). The SCV microenvironment also contains cationic antimicrobial peptides (29), and the addition of PMB (1 μg/mL) to MHB or M9 potentiated both JAV1 and JAV2 growth inhibition. The hydrophobic, fatty acid tail of PMB is required to interact with and disrupt bacterial cell membranes and to stiffen the inner membrane (30). Polymyxin B nonapeptide (PMBN) is a derivative of PMB that lacks the fatty acid tail and cannot lyse membranes or kill bacteria (25, 31). In MHB or M9, PMBN had little or no potentiation effect on JAV1 or JAV2, even at a concentration (30 μg/mL) 12-fold higher than is needed to permeabilize the *S.* Typhimurium outer membrane to the antibiotic novobiocin (21). These data indicated that significant membrane disruption, such as that caused by PMB, is required to enable JAV1 or JAV2 to inhibit bacterial growth.

M9 minimal medium has low levels of nutrients, including calcium (100 nM) and magnesium (1 mM) (32). The concentration of magnesium in M9 is similar to that in the macrophage SCV 1 h after bacterial internalization (33). Potentiation of JAV1 and JAV2 in M9 suggested that limited nutrients or LPS destabilization via low levels of divalent cations increased bacterial vulnerability to the compounds. We found that depletion of divalent cations from M9 by chelation with EDTA (400 μM) further potentiated the growth-inhibitory activities of JAV1 and JAV2, compared to M9 alone. These data demonstrated that the compounds had direct effects on bacteria and likely did not prevent *S.* Typhimurium survival in macrophages solely by acting on the macrophage. Going forward, we used M9 + EDTA medium to study the activities of these compounds in broth culture.

### JAV2 was more rapidly bactericidal than JAV1.

**FIG 3:**
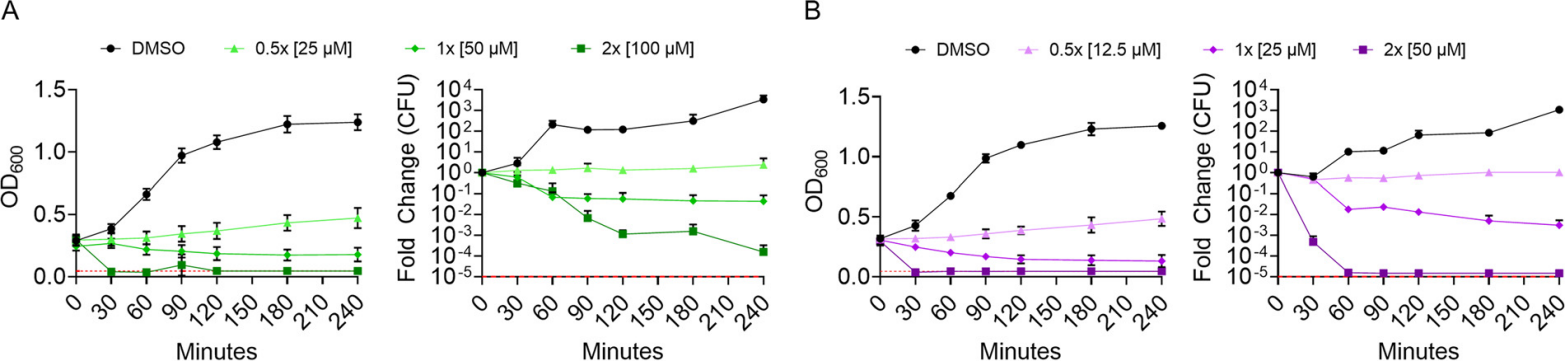
JAV1 and JAV2 are bactericidal. Growth and kill curves of JAV1-treated (A) and JAV2-treated (B) cells are shown. Mid-log-phase *S.* Typhimurium in M9 + 400 μM EDTA (117 μg/mL) was treated with compound and monitored for absorbance (OD_600_ [left]) and CFU (normalized to time zero [right]). Means and SEM of three biological replicates are shown. Red dashed lines represent the limits of detection.

Bacterial growth inhibition could reflect bacterial stasis or death, with or without lysis of cells. To distinguish between these possibilities, we monitored *S.* Typhimurium growth by absorbance and, in parallel, survival by plating for CFU. Bacteria were grown to mid-log phase in M9 + EDTA and exposed to dimethyl sulfoxide (DMSO), JAV1, or JAV2. At subinhibitory concentrations (0.5× MIC_99_), both compounds prevented growth for 4 h (Fig. 3A and B). At inhibitory concentrations (1× or 2× MIC_99_), both compounds reduced absorbance and CFU, suggesting cell lysis and killing, respectively. However, JAV2 was more lethal than JAV1, killing ~9,000 times more cells over the first 60 min at 2× MIC_99_ than JAV1. Thus, under nutrient-limited and LPS-disrupting conditions, both compounds were bactericidal and lysed cells, but JAV2 was considerably more lethal.

### JAV1 and JAV2 decreased membrane electric potential.

**FIG 4:**
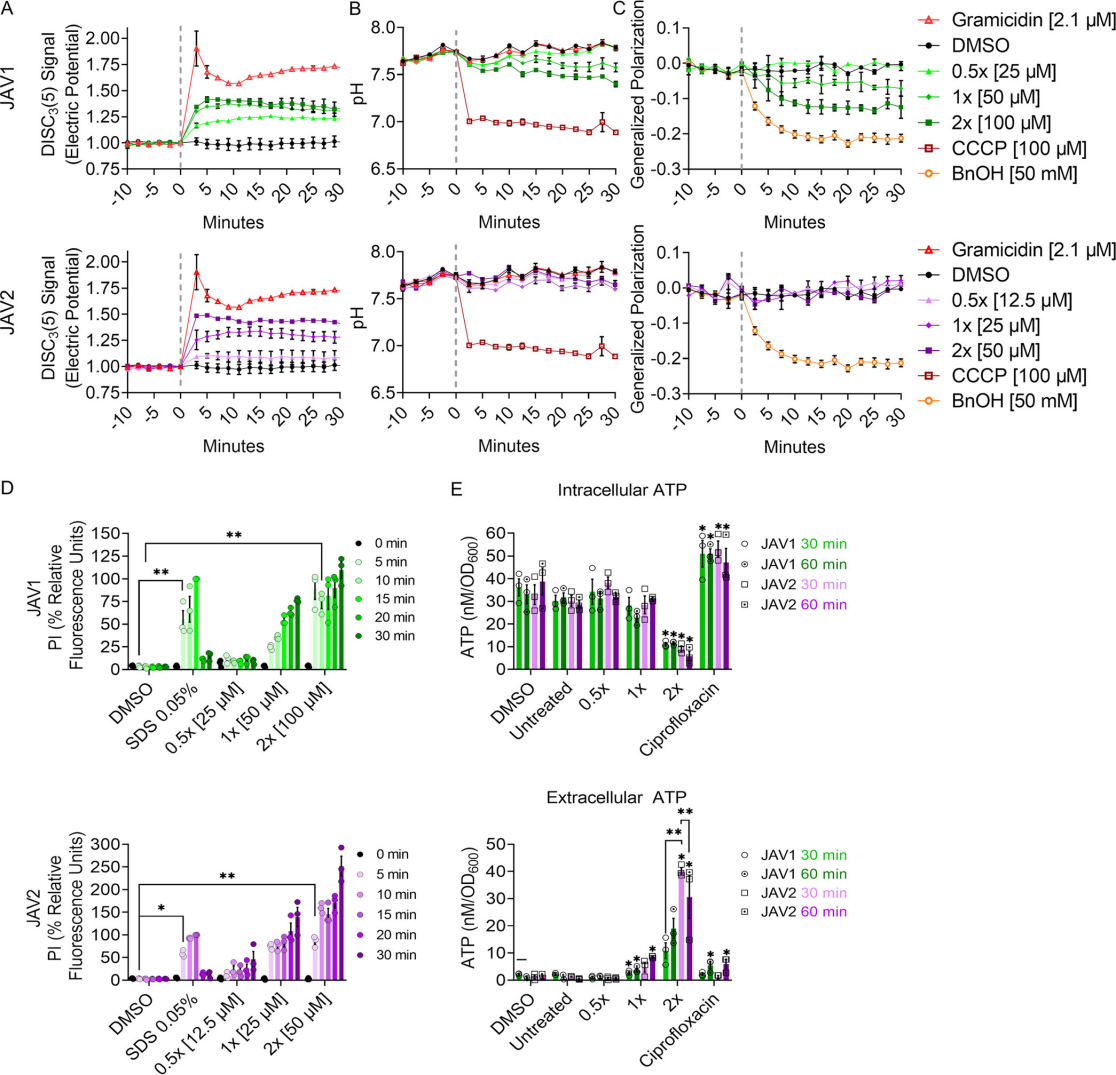
Both JAV1 and JAV2 disrupt electric potential and inner membrane integrity, but only JAV1 disturbs the pH gradient and increases membrane fluidity. (A to C) Mid-log-phase *S.* Typhimurium cultures in M9 + 400 μM EDTA were preloaded with DiSC_3_(5) to monitor membrane electric potential (A), BCECF-AM to measure intracellular pH (B), or Laurdan for membrane fluidity, as indicated by generalized polarization (C). Bacteria were treated as indicated at time zero (vertical gray line) with JAV1 (top) or JAV2 (bottom) and fluorescence was measured for another 30 min. Samples were normalized to time zero (gray vertical line). (D and E) Bacteria were treated at time zero as indicated and evaluated for inner membrane integrity (propidium iodide staining), normalized to SDS at 15 min (D), or intra- (top) and extracellular (bottom) ATP concentrations (luciferase), with each sample normalized to the number of bacteria (OD_600_) (E). For all assays, means and SEM of three biological replicates were performed in technical triplicate. Symbols in panel D: *, *P = *0.01; **, *P* < 0.0001 (determined by two-way ANOVA and Tukey’s multiple comparisons); overall *P* and *F* values and total degrees of freedom were <0.0001, 53.55, and 5, respectively. Symbols in panel E: *, *P = *0.0192; **, *P* <0.001 (determined by two-way ANOVA and Tukey’s multiple comparisons). The overall *P* and *F* values and total degrees of freedom were <0.0001, 70.34, and 5, respectively.

Bacterial cell lysis could occur during or after disruption of proton motive force (PMF), which consists of the transmembrane electric potential and the pH gradient (34). We therefore monitored electric potential with 3,3′-dipropylthiadicarbocyanine iodide [DiSC_3_(5)], which integrates into polarized membranes, where its fluorescence is quenched. Upon perturbation of electric potential, DiSC_3_(5) is expelled from the membrane into the aqueous environment, where it fluoresces brightly (35). Mid-log-phase *S.* Typhimurium cultures were loaded with DiSC_3_(5) and treated with JAV1 or JAV2 (Fig. 4A). As expected, DiSC_3_(5) signal was increased by the ionophore gramicidin (36). Both JAV1 and JAV2 increased DiSC_3_(5) signal in a dose-dependent and sustained manner; the reported values are likely underestimates, since exposure of JAV1 or JAV2 to DiSC_3_(5) in the absence of cells partially quenched fluorescence (see Fig. S3A). Thus, both JAV1 and JAV2 compounds immediately disrupted membrane electric potential.

10.1128/mbio.01790-22.3 FIG S3 JAV1, JAV2, CCCP, and gramicidin quenched DiSC_3_(5) signal, but JAV1 and JAV2 did not alter BCECF-AM signal. The indicated compounds were incubated with DiSC_3_(5) (A) or BCECF-AM (B) in the absence of cells for 10 min prior to measuring fluorescence. Data were normalized to the DMSO control. Download FIG S3, PDF file, 0.1 MB. Copyright © 2022 Villanueva et al. 2022 Villanueva et al. https://creativecommons.org/licenses/by/4.0/ This content is distributed under the terms of the Creative Commons Attribution 4.0 International license.

### JAV2 rapidly disrupted the lipid bilayer, whereas JAV1 increased membrane fluidity.

Disruption of membrane potential could result from damage to the lipid bilayer. Alternatively, if membranes remain intact upon electric potential disruption, bacteria maintain PMF by decreasing cytosolic pH (34). A pH-sensitive, ratiometric dye, 2′,7′-bis-(2-carboxyethyl)-5-(and-6)-carboxyfluorescein, acetoxymethyl ester (BCECF-AM) was used to monitor changes in the cytosolic pH of *S.* Typhimurium in response to JAV1 and JAV2. Mid-log-phase *S.* Typhimurium loaded with BCECF-AM and treated with the protonophore carbonyl cyanide *m*-chlorophenyl hydrazone (CCCP) underwent a rapid decrease in intracellular pH, as expected (Fig. 4B). JAV2 treatment did not alter pH, indicating that this compound disrupts the electric potential and thereby expels DiSC_3_(5) by solubilizing the lipid bilayer. In contrast, JAV1 treatment decreased cytosolic pH steadily over time, suggesting JAV1 enables ion flux across the membrane, and *S.* Typhimurium partially compensates by increasing the cellular concentration of hydrogen ions (H^+^).

Compounds such as JAV1 that disrupt electric potential but enable membranes to remain intact may cause membranes to leak ions by changing membrane fluidity; leakage occurs when more fluid membrane areas interface with more rigid areas (37, 38). We monitored membrane fluidity by calculating Laurdan generalized polarity (GP); Laurdan integrates into membranes and changes the fluorescence emission spectrum when water is nearby (39). Cells were loaded with Laurdan and treated with benzyl alcohol, which fluidized membranes and decreased GP, as expected (Fig. 4C). JAV1 fluidized membranes in a dose-dependent manner at concentrations of 1× MIC_99_ and 2× MIC_99_, but JAV2 did not. While this assay does not distinguish between fluidity changes in the inner versus the outer membrane, it demonstrated that JAV1, but not JAV2, altered membrane structure by decreasing lipid packing.

Disruption of membranes due to lipid solubilization (JAV2) or increased fluidity (JAV1) could suggest failure of the membrane barrier. Barrier function was quantified based on fluorescence associated with intercalation into DNA of the cell-impermeable dye propidium iodide (PI). Fluorescence was monitored before and after compound exposure, and as expected, SDS increased fluorescent signal within 5 min (Fig. 4D). Within 10 and 5 min, respectively, 1× MIC_99_ of JAV1 or JAV2 enabled PI to bind DNA. We also monitored the escape of ATP from the cell with a luciferase-based ATP assay. After 30 min, cells treated with JAV2 at 2× MIC_99_ released significantly more ATP than cells treated with JAV1 (Fig. 4E). Together, these data indicated that JAV2 was more effective at disrupting the inner membrane barrier than JAV1. Altogether, the observations that JAV1 treatment decreased cytosolic pH and increased membrane fluidity, while PI access and ATP release were delayed relative to results after treatment with JAV2, are consistent with the more subtle effects of JAV1 on cell viability. Bacteria appeared to have time to respond physiologically to JAV1 prior to disruption of barrier function and death, whereas JAV2 rapidly disrupted the membrane barrier and killed cells.

### JAV1 and JAV2 interacted with liposomes of varied bacterial lipid composition.

**FIG 5:**
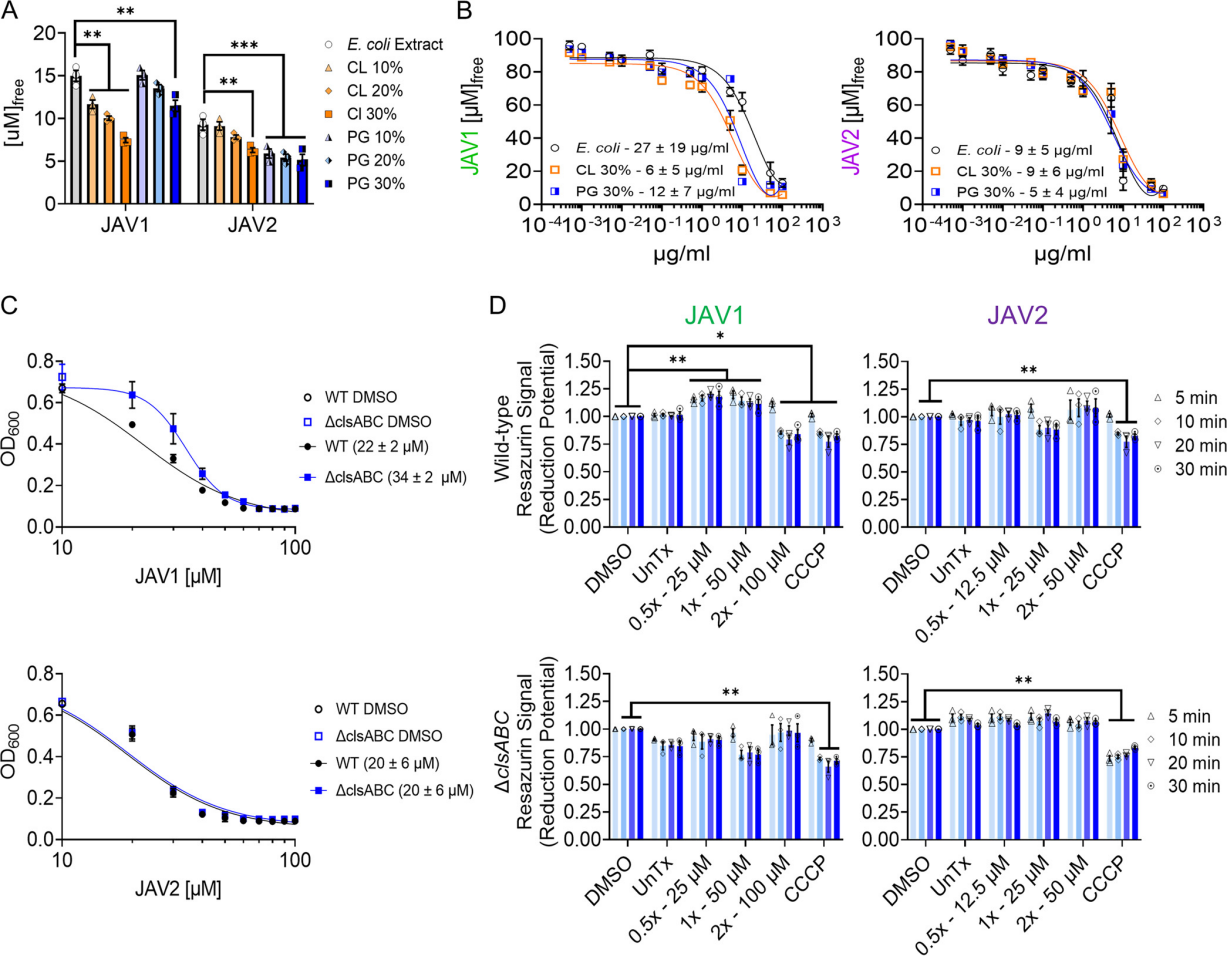
JAV1 and JAV2 interact with liposomes of varied glycerophospholipid composition, but only JAV1 activity partially requires cardiolipin-producing genes and alters reduction potential. (A) A 100 μM concentration of either JAV1 or JAV2 was incubated with liposomes for 10 min and ultracentrifuged, and compound quantities remaining in the supernatant were measured. Liposomes were composed of E. coli total lipid extract (PE, 57.5%; PG, 15.1%; CL, 9.8%; unknown, 17.6%) or supplemented with 10%, 20%, or 30% of either CL or PG glycerophospholipids. Means and SEM of three separate lipid preparations measured in duplicate are shown. **, *P* ≤ 0.001; ***, *P* ≤ 0.0001 (determined by two-way ANOVA and Tukey’s multiple comparisons). The overall *P* and *F* values and total degrees of freedom were <0.0001, 267.1, and 5, respectively. (B) Free JAV1 (left) or JAV2 (right) after incubation with a dose range of liposomes composed of E. coli total lipid extract supplemented with 30% of either CL or PG. Means and SEM of three separate lipid preparations measured in duplicate are shown. Calculated *K_d_* values are indicated under the curves. (C) Growth (based on OD_600_) of wild-type (black) or Δ*clsABC* (blue) *S.* Typhimurium in M9 + 400 μM EDTA exposed to dose ranges of JAV1 (top) or JAV2 (bottom). Means and SEM of three biological replicates performed in technical triplicate are shown. IC_50_ values are indicated in the key in parentheses. (D) Mid-log-phase wild-type (WT; top) or *ΔclsABC* (bottom) *S.* Typhimurium cultures in M9 + 400 μM EDTA were incubated with resazurin (alamarBlue) to quantify membrane reduction potential upon treatment with JAV1 (left) or JAV2 (right). Means and SEM of three biological replicates performed in technical triplicate were normalized to DMSO. *, *P* ≤ 0.002; **, *P* ≤ 0.0001 as determined by two-way ANOVA, and Tukey’s multiple comparisons. The overall *P* and *F* values and total degrees of freedom were as follows: WT and JAV1, <0.0001, 82.38, and 5; WT and JAV2, <0.0001, 39.66, and 5; Δ*clsABC* and JAV1, <0.0001, 11.14, and 5; Δ*clsABC* and JAV2, <0.0001, 18.69, and 5.

Since JAV1 and JAV2 have distinct effects on membrane fluidity and integrity, we determined whether any of the three major bacterial glycerophospholipids or 3-deoxy-d-manno-octulusonic acid (Kdo_2_)-lipid A differentially affected growth in the presence of compound. PE constitutes approximately 75% of the glycerophospholipids in *S.* Typhimurium, PG constitutes ~20%, and CL constitutes ~5% (4). We mixed JAV1 or JAV2 with exogenous glycerophospholipids or Kdo_2_-lipid A, incubated the mixtures with *S.* Typhimurium in M9 + EDTA medium, and monitored growth after 18 h. Of the three glycerophospholipids tested, CL and PG were the most effective at rescuing *S.* Typhimurium growth in the presence of JAV1 or JAV2. However, Kdo_2_-lipid A, which has a higher number of fatty acid tails, rescued the most growth overall (see Fig. S4). Further, fluorescence microscopy with the lipophilic membrane dye FM4-64 showed that JAV1- and JAV2-treated cells accumulated lipid puncta at bacterial poles, where anionic lipids such as CL and PG are located (5, 40) (see Fig. S5). We therefore determined whether JAV1 and JAV2 were preferentially sequestered by either of these glycerophospholipids by using 100-nm liposomes composed of Escherichia coli total lipid extract supplemented with 10%, 20%, or 30% CL or PG. We estimated compound interaction with JAV1 and JAV2 by quantifying UV-visible light absorbance signal from compound remaining in the supernatant after removal of the liposomes by ultracentrifugation. JAV1 was sequestered more efficiently by liposomes containing increased concentrations of CL, and JAV2 was more efficiently sequestered by liposomes containing increased concentrations of PG (Fig. 5A). Nevertheless, both compounds had similar binding affinities between liposomes composed of E. coli total lipid extract or supplemented with 30% CL or PG, as revealed by their *K_d_* (dissociation constant) values (Fig. 5B). We concluded that both JAV1 and JAV2 interacted with and were sequestered by liposomes in a lipid-dependent and lipid-dose-dependent manner with moderate affinity.

10.1128/mbio.01790-22.4 FIG S4 Incubation with phospholipids reduced the effective concentrations of JAV1 and JAV2. Since JAV1 and JAV2 permeabilize inner membranes, we determined whether they interacted with any of the three major bacterial phospholipids or Kdo_2_-lipid A. Concentrations of JAV1 (left) or JAV2 (right) and the indicated lipid (middle) were combined and then incubated with mid-exponential-phase *S.* Typhimurium in M9 + 400 μM EDTA. Growth (OD_600_) was monitored after 18 h and normalized to treatment with DMSO. Green, maximal growth; red, 0% growth. (A) Phosphatidylethanolamine (PE); (B) phosphatidylglycerol (PG); (C) Kdo_2_-lipidA; (D) cardiolipin (CL). The charge, c[log(P)], and structure of each lipid are shown. These lipids vary in size, charge, lipophilicity, and their spatial distribution within Gram-negative bacteria (G. F. Ames, J Bacteriol 95:833–843, 1968, https://doi.org/10.1128/jb.95.3.833-843.1968; C. Sohlenkamp and O. Geiger, FEMS Microbiol Rev 40:133–159, 2016, https://doi.org/10.1093/femsre/fuv008; X. Wang, P. J. Quinn, and A. Yan, Biol Rev Camb Philos Soc 90:408–427, 2015, https://doi.org/10.1111/brv.12114; W. Dowhan, Annu Rev Biochem 66:199–232, 1997, https://doi.org/10.1146/annurev.biochem.66.1.199). PE and PG are largely distributed across the cell axis of both the inner and outer membranes, with higher concentrations of PG near cell poles, while CL is primarily localized to the cell poles and the inner membrane (G. Laloux and Jacobs-Wagner, J Cell Sci 127:11–19, 2014, https://doi.org/10.1242/jcs.138628; R. Arias-Cartin, S. Grimaldi, P. Arnoux, B. Guigliarelli, and A. Magalon, Biochim Biophys Acta 1817:1937–1949, 2012, https://doi.org/10.1016/j.bbabio.2012.04.005; V. A. M. Gold, A. Robson, H. Bao, T. Romantsov, et al., Proc Natl Acad Sci U S A 107:10044–10049, 2010, https://doi.org/10.1073/pnas.0914680107; K. Matsumoto, Mol Microbiol 39:1427–1433, 2001, https://doi.org/10.1046/j.1365-2958.2001.02320.x). Means from three biological replicates are shown. At a concentration of 0.5 μM, all phospholipids tested enabled growth in the presence of JAV1 and JAV2, suggesting the compounds interact with lipids generally. However, JAV1 was more sensitive to lipid negative charge and hydrophobicity, as more growth rescue occurred at lower concentrations of PG and CL compared to that with PE and Kdo2-Lipid A. These data suggest that JAV1 has a bias for anionic lipids with high lipophilicity values, whereas JAV2 appears to interact with phospholipids more generally. Download FIG S4, PDF file, 0.6 MB. Copyright © 2022 Villanueva et al. 2022 Villanueva et al. https://creativecommons.org/licenses/by/4.0/ This content is distributed under the terms of the Creative Commons Attribution 4.0 International license.

10.1128/mbio.01790-22.5 FIG S5 Prolonged JAV1 or JAV2 treatment increased lipid density at cell poles. To establish whether the compounds altered lipid distribution around the bacterial cell, we exposed cells to JAV1 or JAV2 and then fixed and stained with FM 4-64, a cell membrane stain that correlates with lipid density (J. Pogliano, N. Osborne, M. D. Sharp, A. A.-D. Mello, et al., Mol Microbiol 31:1149–1159, 1999, https://doi.org/10.1046/j.1365-2958.1999.01255.x; I. Fishov and C. L. Woldringh, Mol Microbiol 32:1166–1172, 1999, httsp://doi.org/10.1046/j.1365-2958.1999.01425.x; D. I. Cattoni DI, Fiche J-B, Valeri A, Mignot T, Nöllmann M, PLoS One 8:e76268, 2013, https://doi.org/10.1371/journal.pone.0076268). Confocal microscopy images were batch analyzed using the MicrobeJ ImageJ plug-in (A. Ducret, E. M. Quardokus, and Y. V. Brun, Nat Microbiol 1:16077, 2016, https://doi.org/10.1038/nmicrobiol.2016.77) for local intensity events around the perimeter of 1,000 randomly selected cells. (A) Representative, zoomed-in micrographs of *S.* Typhimurium (red) treated as indicated. (B) Example foci determination (blue border) from the cell labeled with an asterisk in the JAV1 2 × 90 min image. Intensity events were considered foci if local fluorescent intensity was above a set threshold and had a diameter of 24 to 48 nm^2^, which is the average size of foci noted in control and sample images ± the standard deviation (12 nm^2^). Raw data (left) were masked for bacteria (middle; red outline trace). Foci were determined as described above (right, blue, and red traced circles). The funnel plots include only foci that were within the bacterial outline and of the appropriate size (small blue circles; the red circled foci were too small). (C) Funnel plots of foci relative to the cell axis. In each condition, the cell perimeter was averaged and normalized to an ideal rod-shaped bacterial length and width. Percent distribution of foci ranged from −15% to +15% for ease of visualization around bacterial perimeter but should be considered absolute values relative to the cell axis for interpretation. Means of 3 biological replicates performed in duplicate are shown. These data indicate that *S.* Typhimurium responds to JAV2 and to a lesser extent JAV1 treatment by increasing lipid abundance at the cell poles. Download FIG S5, PDF file, 1.7 MB. Copyright © 2022 Villanueva et al. 2022 Villanueva et al. https://creativecommons.org/licenses/by/4.0/ This content is distributed under the terms of the Creative Commons Attribution 4.0 International license.

### JAV1 activity partially required CL-producing genes and altered reduction potential.

Since JAV1 sequestration in liposomes correlates with CL, we created genetic knockouts of each of the CL-producing genes in *S.* Typhimurium, *clsA*, *clsB*, and *clsC*. We chose to delete CL-producing genes instead of PG-producing genes because genetic deletions of PG-producing genes are lethal in Gram-negative bacteria without compensatory mutations that reduce the abundance of Braun’s lipoprotein in the cell wall, which requires PG for attachment to the outer membrane (41–43). Single and double CL gene knockouts had no apparent effect on *S. Typhimurium* susceptibility to JAV1, consistent with the functional redundancy of the CL-producing genes: ClsA, ClsB, and ClsC all catalyze *trans*-phosphatidylation reactions between two glycerophospholipids, either two PGs (ClsA and ClsB) or PG with PE (ClsC) to form CL (see Fig. S6). In contrast, the *ΔclsABC* triple mutant strain was more resistant to JAV1, as revealed by a 1.5-fold increase in IC_50_ value compared to wild type (Fig. 5C). The *ΔclsABC* triple mutant strain produced little to no CL (see Fig. S6D). Since CL-protein interactions participate in the bacterial respiratory chain process, we quantified *S.* Typhimurium reduction potential using resazurin (44, 45). As anticipated, treatment with CCCP decreased reduction potential (Fig. 5D). Treatment with JAV2 had no significant effect. However, in a *ΔclsABC-*dependent manner, JAV1 treatment increased reduction potential at 0.5× and 1× MIC_99_ and decreased reduction potential at 2× MIC_99_. These data indicated that JAV1 interacted with CL across a concentration range: at lower JAV1 concentrations, cells responded by increasing respiration, whereas at higher concentrations cells lost their viability.

10.1128/mbio.01790-22.6 FIG S6 *S*. Typhimurium cardiolipin (CL) synthesis. (A to C) *S*. Typhimurium encodes three CL synthases, *clsA*, *clsB*, and *clsC* (M. B. Cian, J. A. Mettlach, A. E. Zahn, N. P. Giordano, et al., Microbiol Spectr 10:e02617-21, 2022, https://doi.org/10.1128/spectrum.02617-21; Z. D. Dalebroux, M. B. Edrozo, R. A. Pfuetzner, S. Ressl, et al., Cell Host Microbe 17:441–451, 2015, https://doi.org/10.1016/j.chom.2015.03.003). (A) ClsA and ClsB catalyze the transfer of a phosphatidyl group from one phosphatidylglycerol (PG) molecule to a second PG molecule, resulting in glycerol and diphosphatidylglycerol, which is also known as cardiolipin. (B) *clsB* has a broader substrate specificity than *clsA* or *clsC* and catalyzes synthesis of phosphatidyltrehalose, diphosphatidyltrehalose, phosphatidylalcohols, and PG though transphosphatidylation. (C) ClsC also catalyzes a transphosphatidylation reaction between one phosphatidylethanolamine (PE) and one PG molecule, which generates ethanolamine and CL. (D) *S.* Typhimurium (SL1344) Δ*clsABC* has less cardiolipin than the wild type. Two-dimensional thin-layer chromatography (TLC) analysis of total membrane extract of wild-type and Δ*clsABC* mutant *S.* Typhimurium is shown. The empty circle denotes the predicted migration position of CL in the mutant *ΔclsABC* extract. Membrane extracts were visualized by iodine vapor, and CL was identified using a commercial standard (Avanti). (E) JAV1 has no apparent effect on single or double *clsA*, *clsB*, or *clsC* mutant strains. Single and combination double knockouts of *clsA*, *clsB*, or *clsC* in *S.* Typhimurium were treated with JAV1 as indicated. Absorbance (OD_600_) values were normalized to the DMSO control (100%). Means and SEM of three biological replicates performed in technical triplicate are shown. Download FIG S6, PDF file, 0.5 MB. Copyright © 2022 Villanueva et al. 2022 Villanueva et al. https://creativecommons.org/licenses/by/4.0/ This content is distributed under the terms of the Creative Commons Attribution 4.0 International license.

### Host cells exhibited minimal stress responses at concentrations of JAV1 and JAV2 that enabled killing of *S.* Typhimurium in macrophages.

**FIG 6:**
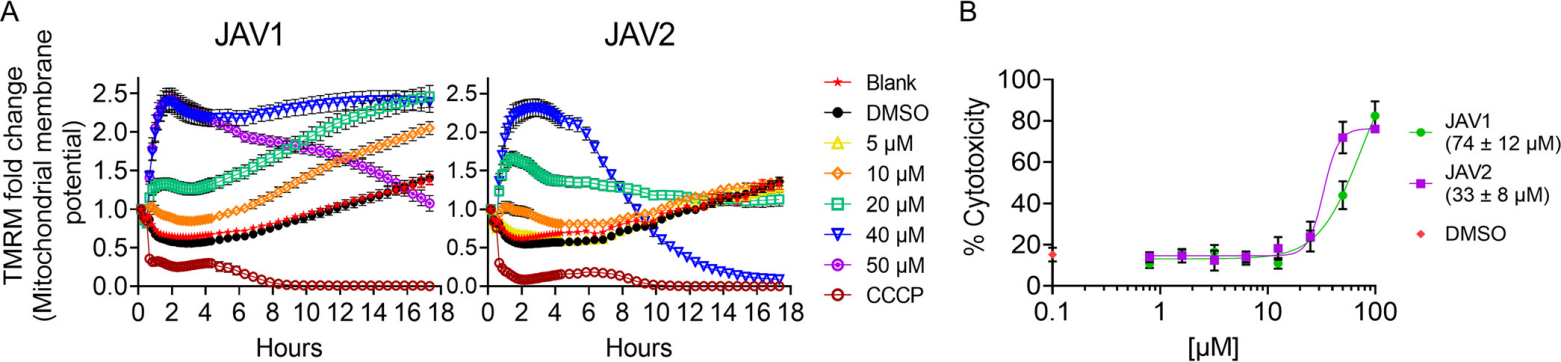
Mitochondrial membranes resisted JAV1 and JAV2 at concentrations that kill *S.* Typhimurium in macrophages. (A) RAW 264.7 cells were loaded with the mitochondrial membrane potential indicator dye TMRM and treated as indicated with JAV1 (left) or JAV2 (right). Fluorescence was quantified at 10-min intervals for the first 4.5 h and at 30-min intervals for the remainder of the experiment. Means and SEM of three biological replicates performed in technical triplicate are shown (see also Fig. S7 in the supplemental material). Data were normalized to time zero. (B) Supernatant accumulation of LDH was quantified from RAW 264.7 cells treated as indicated with JAV1 (green) or JAV2 (purple). Percent cytotoxicity was normalized to that in lysed cells. Means and SEM of three biological replicates performed in technical duplicate are shown. The 50% cytotoxic concentrations (CC_50_s) are indicated in the key in parentheses.

Mitochondrial membranes have glycerophospholipid contents similar to that of bacteria, including the presence of PG and CL in both membranes (46, 47). To assess whether JAV1 or JAV2 perturbed mitochondria, we incubated macrophages with tetramethylrhodamine, methyl ester (TMRM), a fluorescent indicator of mitochondrial membrane voltage. At concentrations that reduced *S.* Typhimurium load within macrophages (<10 μM), JAV1 and JAV2 had little effect (Fig. 6A and Fig. S7). At higher concentrations, the compounds caused rapid and sustained mitochondrial membrane hyperpolarization, an indicator of stress. JAV1 or JAV2 could affect mitochondria directly or indirectly by disrupting host cell plasma membrane integrity, which causes calcium ion influx and is detrimental to the cell (48, 49). We monitored plasma membrane damage upon exposure to JAV1 or JAV2 by quantifying the release of lactate dehydrogenase (LDH) into the medium (50). Treatment with less than 10 μM JAV1 or JAV2 did not increase LDH release above that of vehicle, but higher concentrations significantly permeabilized host cell membranes (Fig. 6B). We concluded that high concentrations of compound could indirectly cause hyperpolarization of mitochondrial inner membranes via loss of host plasma membrane integrity. Nevertheless, at JAV1 and JAV2 concentrations that kill *S.* Typhimurium in macrophages, mitochondrial and host cell membranes were minimally affected.

10.1128/mbio.01790-22.7 FIG S7 Mitochondrial membranes resist JAV2 at concentrations that kill *S.* Typhimurium in macrophages. Representative micrographs are from data in Fig. 6A for JAV2. Download FIG S7, PDF file, 0.6 MB. Copyright © 2022 Villanueva et al. 2022 Villanueva et al. https://creativecommons.org/licenses/by/4.0/ This content is distributed under the terms of the Creative Commons Attribution 4.0 International license.

### JAV1 and JAV2 accumulated in macrophage acidic compartments.

**FIG 7:**
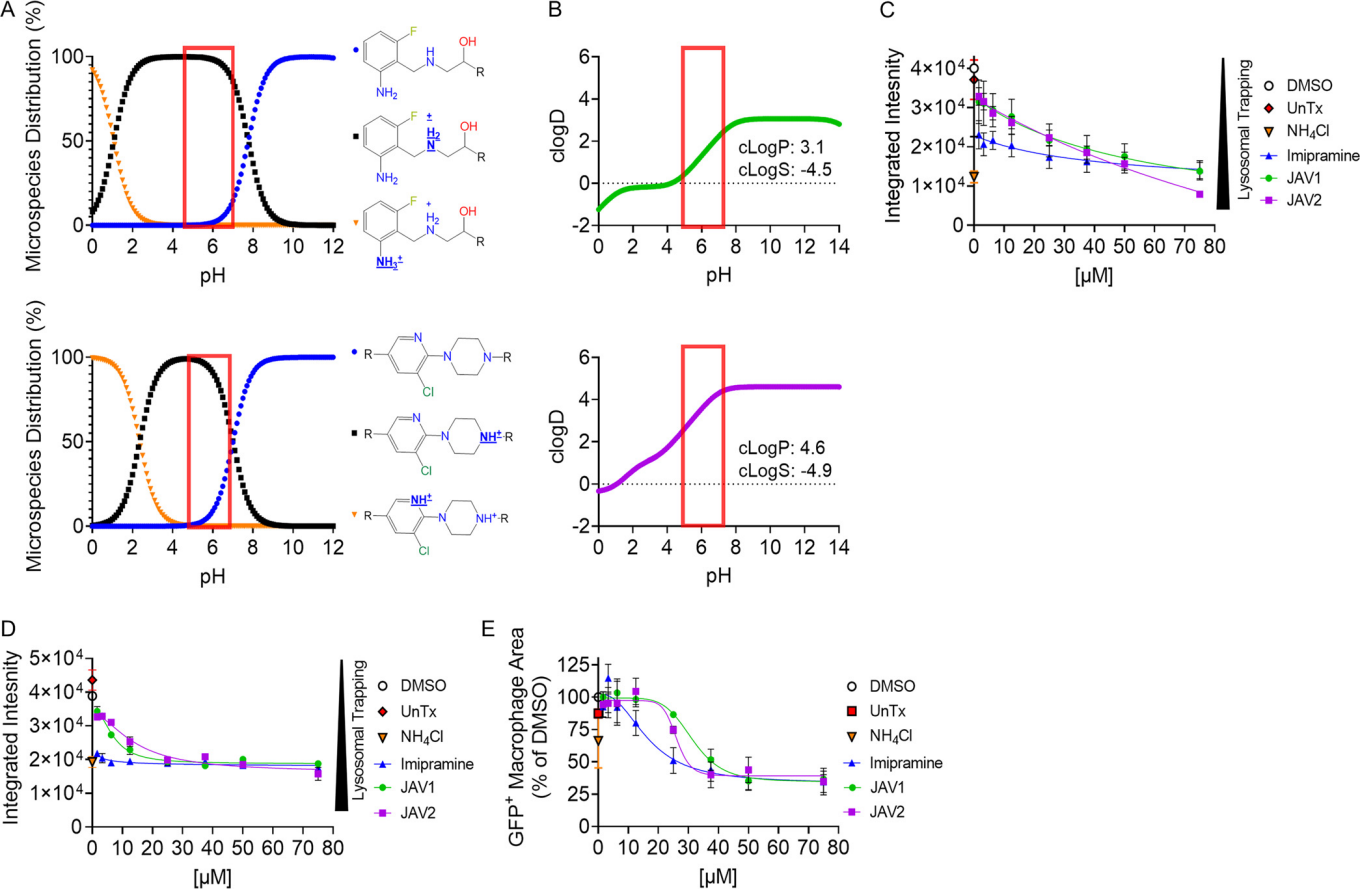
JAV1 and JAV2 are trapped within acidic compartments and phagosomes in macrophages. (A and B) ChemAxon-predicted microspecies distributions of JAV1 (top) and JAV2 (bottom) as a function of pH (A) and predicted partitioning of JAV1 (top) or JAV2 (bottom) between lipid and aqueous phases (B), determined by the c[log(D)] versus pH. The c[log(P)] (lipophilicity) and c[log(S)] (solubility) values for pH 7.4 are listed under the curves. The red box indicates the pH of the *S.* Typhimurium-containing phagosome. (C to E) RAW264.7 cells were uninfected (C) or infected with *S.* Typhimurium harboring a chromosomal *sifB*::*gfp* reporter (D and E). Cells were treated with DMSO, ammonium chloride (NH_4_Cl [10 mM] to prevent vesicle acidification), or the indicated concentrations of imipramine (positive control), JAV1, or JAV2. Cells were quantified for LysoTracker Red DND-99 fluorescence within macrophages, defined as the integrated intensity of LysoTracker across macrophage area. (C) Uninfected cells were treated for 1 h before imaging. Means and SD of technical duplicates performed in biological duplicate are shown. (D and E) Infected cells were treated at 4.5 h after infection for 1.5 h prior to imaging. Images were quantified for LysoTracker signal (D) and GFP^+^ (E) macrophage area. Means and SEM of three biological replicates performed in technical duplicate are shown.

One mechanism by which host cell membranes could be protected from JAV1 and JAV2 is by their rapid sequestration within acidic vesicles, a process known as lysosomal trapping (19, 51). Both compounds have ionizable amine groups that are predicted to become protonated near pH 6, increasing compound solubility and trapping the compounds within acidic vesicles, such as the SCV (Fig. 7A and B). To establish whether JAV1 or JAV2 accumulated in acidic macrophage vesicles, we used LysoTracker Red DND-99 (LTR), a weak base that accumulates in low-pH vesicles and fluoresces. LTR fluorescent intensity declines as vesicles basify upon treatment of cells with ammonium chloride (NH_4_Cl), for instance. Similarly, treatment of cells with imipramine modestly basifies vesicles and reduces LTR signal (19, 51, 52). Cells were untreated or treated for 1 h with DMSO, imipramine, NH_4_Cl (10 mM), JAV1, or JAV2. Cells were then incubated with LTR for 1 to 1.5 h prior to imaging. As expected, untreated and DMSO-treated cells accumulated LTR, and treatment with NH_4_Cl or imipramine reduced LTR signal (Fig. 7C). Both JAV1 and JAV2 reduced LTR signal in a dose-dependent manner, indicating they underwent lysosomal trapping.

To establish whether JAV1 and JAV2 lysosomal trapping correlated with decreased *S.* Typhimurium load, we performed similar experiments on macrophages infected with *S.* Typhimurium *sifB*::*gfp.* At 4.5 h after infection, cells were treated with JAV1 or JAV2 for 1.5 h and then monitored for GFP^+^ pixels within macrophage areas and LTR signal (Fig. 7D and E). As expected, LTR and GFP signals were higher in untreated and DMSO-treated cells than in NH_4_Cl- or imipramine-treated cells, as basifying vesicles with NH_4_Cl or treating macrophages with imipramine reduces the survival of intracellular bacteria (21, 53, 54). JAV1 and JAV2 treatment decreased LTR and bacterial GFP signals in a dose-dependent manner. Together, these results suggest that JAV1 and JAV2 accumulate within acidic vesicles of macrophages, indicating they could be targeted to *S.* Typhimurium SCVs during infection. Lysosomal trapping could help account for host membrane resistance to JAV1 and JAV2 and *S.* Typhimurium sensitivity to the compounds during infection, compared to in broth.

### JAV1 and JAV2 significantly prolonged survival of infected Galleria mellonella larvae.

**FIG 8:**
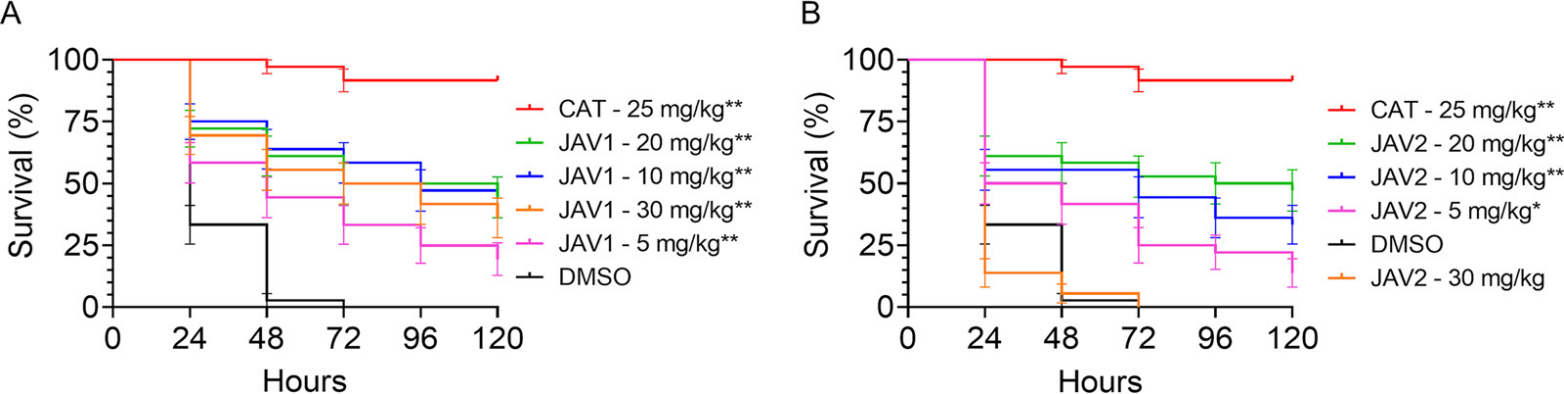
Treatment with JAV1 or JAV2 prolonged survival of infected G. mellonella larvae. Percent survival of G. mellonella larvae infected on day 0 with 1.7 × 10^5^ CFU/larva and treated as indicated with DMSO, chloramphenicol (CAT), JAV1 (A), or JAV2 (B). Larvae were monitored for survival over 5 days. Means and SEM are shown for three independent experiments with *n* = 12 larvae per condition. **, *P < *0.0001, as determined by log-rank (Mantel-Cox) test.

Insect and mammalian cellular innate immunity are highly conserved (55–57), and *S.* Typhimurium resides within hemocytes in Drosophila melanogaster (58). Larvae of the wax moth Galleria mellonella engulf and eliminate invading bacteria in hemocytes, in which the phagosomes undergo an acidification process similar to that of mammalian cells (59, 60). Therefore, we asked whether JAV1 and JAV2 improved survival of G. mellonella larvae infected with *S.* Typhimurium (61, 62). Larvae were infected with approximately 1.7 × 10^5^ CFU, treated with vehicle, chloramphenicol, or compound, and monitored for survival over the course of 5 days (Fig. 8 and Fig. S8). JAV2 appeared to be toxic to the larvae at the highest dose of 30 mg/kg. Nevertheless, both compounds significantly prolonged the lives of infected G. mellonella larvae in a dose-dependent manner compared to DMSO treatment. Thus, JAV1 and JAV2 have *in vivo* antimicrobial efficacy in the G. mellonella model of infection.

10.1128/mbio.01790-22.8 FIG S8 Controls for survival of Galleria mellonella larvae infection experiments. (A) Percent survival of larvae infected with 1.7 × 10^5^ CFU/larva over a 5-day time course. Infection and treatment controls included the following: handling only, double needle stick, mock infection, mock infection with DMSO treatment, infection, infection with DMSO treatment, and infection with chloramphenicol (CAT) treatment. Means and SEM of three biological replicates performed in technical triplicate are shown, with *n* = 12 larvae per condition. **, *P < *0.0001 as determined by log-rank (Mantel-Cox) test. (B) JAV1 and JAV2 conversions from milligrams per kilogram to micromolar. Download FIG S8, PDF file, 0.1 MB. Copyright © 2022 Villanueva et al. 2022 Villanueva et al. https://creativecommons.org/licenses/by/4.0/ This content is distributed under the terms of the Creative Commons Attribution 4.0 International license.

## DISCUSSION

JAV1 and JAV2 are active against *S.* Typhimurium within macrophages and in an insect larvae infection model. These small molecules have antibacterial activity against *S.* Typhimurium under broth conditions that mimic infection and that cause damage to the outer membrane. JAV1 and JAV2 have distinct destructive effects on bacterial inner membranes, and both compounds are bactericidal. While they are too toxic to mammalian cells to become lead compounds, their study highlights useful chemical attributes for developing new classes of antimicrobials for intravesicular pathogens. Below, we discuss JAV1 and JAV2 mechanisms of action and the potential utility of lipophilic molecules that exploit lysosomal trapping and soluble innate immunity in the development of anti-infectives.

### Proposed antibacterial action of JAV1: increased membrane fluidity.

The interface of fluid and rigid membrane areas allows for leakage of cytosolic contents from the cell and entry of amphipathic, hydrophilic peptides, and even large molecules, such as plasmid DNA (63–66). We therefore posit that after JAV1 crosses a damaged LPS layer, it integrates with the glycerophospholipids of the outer and inner membranes, increasing membrane fluidity and disrupting electric potential across the inner membrane. The bacterial cell appears to respond to the change in electric potential by decreasing cytoplasmic pH; nevertheless, the cell loses membrane integrity within 10 min of JAV1 treatment. Lipid sequestration, binding affinity, and genetic interaction data support that JAV1 preferentially interacts with negatively charged lipids and partially relies on CL for antimicrobial activity. CL-protein interactions contribute to the function of bacterial respiration complexes (44, 67), and their disruption may account for the changes in reduction potential observed with JAV1 treatment. In addition, or alternatively, the observed JAV1 disruption in membrane potential could alter the localization of membrane proteins that require active membrane potential for proper localization and function, such as nitrate reductase complexes and the cell division machinery proteins MinD, MreB, and FtsZ (68, 69). Other antimicrobials that disrupt membrane potential and inhibit cellular respiration (e.g., rhodomyrtone, antimicrobial peptides) similarly cause cell death over time (65, 70–72). In summary, JAV1 interacts with bacterial lipids with some degree of specificity for CL, disrupts membrane electric and reduction potentials, and decreases membrane integrity, likely killing bacteria or enabling their killing by macrophages.

### Proposed antibacterial action of JAV2: inner membrane disruption.

After reaching the inner membrane, JAV2 immediately disrupts membrane electric potential without causing a decrease in cytoplasmic pH. Instead, JAV2 rapidly permeabilizes the inner membrane and kills bacteria. JAV2 has a moderate affinity for lipids, with no detectable effect on respiration. JAV2 could therefore have a generalized disrupting effect on membrane proteins due to alterations of the membrane architecture. This is supported by the observed JAV1- and JAV2-induced membrane distortion events near the cell poles. In summary, JAV2 interacts with lipids to disrupt membrane electric potential and rapidly reduces membrane integrity, activities that likely enable macrophages to quickly eliminate the bacteria.

### The potential to exploit lysosomal trapping to concentrate antimicrobials at the site of intravesicular pathogens.

During infection, JAV1 and JAV2 accumulate in low-pH vesicles, including acidified SCVs, in a dose-dependent manner. The compounds may reach the SCV in a variety of ways: diffusion through the cytosol, integration into the host cell membrane, and/or capture by endocytosis followed by trafficking to acidified cellular compartments. In low-pH vesicles, the compounds become trapped in the lumen and could be delivered to SCV during phagolysosome fusion, which is required for *S.* Typhimurium infection. It cannot be ruled out that in macrophages, JAV1 and JAV2 contribute to bacterial killing by an unknown mechanism(s) that acts on the pathogen and/or the host cell. However, we postulate that the accumulation of JAV1 and JAV2 within the SCV contributes to their efficacy during infection. These observations highlight the potential use of weak base antimicrobial structures to target vesicular pathogens, including not only Salmonella enterica species but also Coxiella burnetii (73), Legionella pneumophila (74), Brucella species (75, 76), Mycobacterium species (77–79), and eukaryotic parasites such as *Leishmania* (80, 81).

### The potential to exploit innate immunity to enable small molecules to cross the outer membrane during infection.

Conditions that weaken the outer membrane barrier and enable JAV1 and JAV2 to inhibit bacteria, including nutrient limitation and physical damage to the outer membrane, are characteristic of phagosomes. Of note, the addition of EDTA or PMB to M9 minimal medium was considerably more effective at enabling the compounds to inhibit growth than the addition of PMBN. While EDTA, PMB, and PMBN all compromise the LPS layer and are antimicrobial adjuvants (82–84), EDTA is better at potentiating lipophilic antimicrobials in Gram-negative bacteria than PMBN, as observed here and with the human and canine antimicrobial robenidine (85). For PMB, the fatty acid tail enables it to physically disrupt the membranes in a manner that PMBN, lacking the tail, cannot (30, 31). It therefore appears that significant disruption to LPS, such as that caused by the removal of divalent cations by EDTA or by PMB, is needed to enable JAV1 and JAV2 to quickly breach the outer membrane. We speculate that during infection, a combination of low levels of divalent cations and accumulation of cationic antimicrobial peptides in the SCV potentiates JAV1 and JAV2 by destabilizing LPS sufficiently to enable the compounds to traverse the outer membrane and inhibit virulence. Whether the compounds themselves or in conjunction with host innate immunity kill the bacteria during infection remains unknown. Nevertheless, exploiting innate immunity to enable small-molecule access to Gram-negative bacteria or to eliminate a pathogen damaged by small molecules appears to be a viable antimicrobial approach.

### The potential utility of lipophilic compounds to treat infections.

The lipophilic character of JAV1 and JAV2 may contribute to their efficacy in macrophages and in wax worms, as their lipophilicity likely enables them to exploit host-induced outer membrane damage to target inner membranes. Lipinski’s “Rule of Five” for drugs does not favor lipophilicity (86). However, existing antibiotics are recognized as outliers to these rules, and lipophilic antibiotics should not be deprioritized based on violation of two or more of these rules (87). Moreover, others have argued that lipophilic compounds have distinct advantages in the treatment of severe acute bacterial infections (88, 89). Lipophilic antimicrobials are maintained more easily in body tissues, while hydrophilic antimicrobials concentrate more in plasma (90). Critically ill septic patients often have damage to endothelia and increased capillary leakage, which shifts fluid from body tissue to the interstitial space, diluting the plasma concentration of hydrophilic antibiotics and requiring higher and potentially toxic doses of hydrophilic antimicrobials for treatment (91, 92). In contrast, lipophilic antimicrobials tend to diffuse more slowly from tissues upon capillary leakage and maintain their efficacy without the need for toxic doses (90, 93). Despite these advantages, current lipophilic antibiotic therapies are imperfect at treating intracellular infections. Although lipophilic antibiotics penetrate and accumulate in cells, they are not always efficacious (89, 94) and instead depend on the physicochemical microenvironment of the infection and synergy with host defenses (88, 95, 96). Moreover, lipophilic antibiotics typically target actively growing intracellular bacteria in lieu of nongrowing or persister cell types that require a minimum level of active PMF and ATP metabolism (97–100). Thus, intracellular infections often require single or combination antibiotic therapies for extended periods, which can be detrimental to patients and increase the development of resistant bacteria (2, 72, 101–103). We argue that future antimicrobial development should take advantage of chemicals that interrupt bacterial infection by exploiting host innate immunity to target the bacterial inner membrane.

### Conclusions.

The observations presented in this work substantiate the importance of the exploration of new chemical scaffolds that have antimicrobial activity in a host-pathogen-relevant context. They also highlight amine substitution of lipophilic antimicrobials to exploit vesicular pathogen pathophysiology and the potential for exploitation of the host innate immune system to access and target intracellular Gram-negative bacterial pathogens. Finally, the data provide additional support for the idea that bacterial inner membranes are a feasible target for novel anti-infective agents.

## MATERIALS AND METHODS

### Reagents.

JAV1 was identified as DET 021(13){IUPAC 1-[(2-amino-6-fluorophenyl)methylamino]-3-(3,4-dichlorophenoxy)propan-2-ol; SMILES NC1=CC=CC(F)=C1CNCC(O)COC=CC=C(Cl)C(Cl)=C1; May bridgecode RDR03027} and JAV2 was identified as DET 031(13) {IUPAC 3-((4-[3-chloro-5-(trifluoromethyl)-2-pyridinyl]piperazino)methyl)-1H-indole); SMILES FC(F)(F)C =CN=C(N2CCN(CC =CNC =C3C=CC=C4)CC2)C(Cl)=C1; Maybridge code HTS04862}. Both compounds were resuspended in DMSO at 20 mM and stored at room temperature. CCCP (Sigma catalog number C2759), gramicidin (Sigma catalog number G5002), polymyxin B sulfate (Sigma catalog number P1004), and polymyxin B nonapeptide (Sigma catalog number P2076) were solubilized just prior to use.

### Bacterial strains and media.

For initial screening and lysosomal trapping imaging experiments, macrophages were infected with Salmonella enterica serovar Typhimurium strain SL1344 (*sifB*::*gfp*, CSD1021) (13, 104). All other experiments used wild-type *S.* Typhimurium (SL1344, CSD001) (105) or SL1344 Δ*clsABC* (JAV1283). JAV1283 was generated from ST14028s Δ*clsABC* (106) by successive P22 phage transductions. Briefly, lysate was prepared by adding 100 μL of 10^8^ PFU/mL phage stock to 3.9 mL of Luria-Bertani (LB) broth (107, 108). One milliliter of LB-grown overnight ST14028s Δ*clsABC* culture was added and the mixture was incubated overnight at 37°C with aeration. The mixture was transferred to a 15-mL conical tube, 100 μL of chloroform was added, and the mixture was incubated for exactly 5 min at 37°C with aeration. The culture was centrifuged at maximum speed at 4°C for 20 min. Supernatants were transferred to a fresh conical tube, and 200 μL of chloroform was added for storage at 4°C. The recipient SL1344 streptomycin (STR)-resistant strain was grown overnight, and 100 μL of cells was combined with 100 μL of donor phage lysate, incubated for 30 min at 37°C with aeration, and plated onto STR with antibiotics to select for transduction. Transductants were diluted into 20 μL phosphate-buffered saline (PBS) and struck for single colonies to verify selection and dilute the phage.

Unless otherwise stated, prior to experiments bacteria were grown overnight, ~18 h, at 37°C with aeration in cation-adjusted Mueller-Hinton broth (Sigma catalog number 90922) (109) with 30 μg/mL STR. Kanamycin (KAN; 30 μg/mL) was included for the *sifB*::*gfp* strain. Tetracycline (TET; 10 μg/mL), chloramphenicol (CAT; 30 μg/mL), and KAN were included for the SL1344 Δ*clsABC* strain. To obtain mid-log-phase cells, overnight cultures were diluted 1:100 in M9 minimal medium (42 mM Na_2_HPO_4_, 22 mM KH_2_PO_4_, 18.7 mM NH_4_Cl, 8.6 mM NaCl, 0.1% Casamino Acids, 1 mM MgSO_4_, 0.4% glucose), pH 7.2, supplemented with 400 μM EDTA (Sigma catalog number E9884) (32), and then grown at 37°C with aeration to mid-log phase (optical density at 600 nm [OD_600_] of 0.4 to 0.6).

### SAFIRE assay and analysis.

As previously described (13), RAW 264.7 macrophages (between passages 1 and 6) were grown in complete Dulbecco’s modified Eagle’s medium (DMEM) to approximately 70 to 90% confluence, scraped, washed, resuspended, diluted to 5 × 10^4^ in 100 μL, and seeded in black, 96-well, glass-bottomed plates (Brooks Life Sciences catalog number MGB096-1-2-LG-L) at 37°C with 5% CO_2_. Twenty-four hours later, bacteria grown overnight in LB were diluted into 50 μL PBS and added to a final concentration of 1 × 10^7^ *S.* Typhimurium SL1344 (*sifB*::*gfp*)/mL for an approximate multiplicity of infection of 30 bacteria per macrophage cell. Forty-five minutes after bacterial addition, gentamicin (Sigma catalog number G1264) was added to a final concentration of 40 μg/mL to inhibit the growth of remaining, extracellular bacteria. Two hours after infection, gentamicin-containing medium was removed and replaced with 200 μL fresh DMEM with compound or DMSO (Sigma catalog number 276855) to the stated final concentrations. At 17.5 h after infection, PBS containing MitoTracker Red CMXRos (Life Technologies catalog number M7512), a vital dye for mitochondrial electric potential, was added to a final concentration of 100 nM. At 18 h after infection, 16% paraformaldehyde was added to a final concentration of 4% and incubated at room temperature for 15 min. Cells were washed twice with PBS and stained for 20 min with 1 μM 4′,6-diamidino-2-phenylindole (DAPI) and stored in 90% glycerol in PBS until imaging. Samples were imaged in six fields of view per well using a semiautomated Yokogawa CellVoyager CV1000 confocal scanner system with a 20×, 0.75-numerical aperture (NA) objective. A MATLAB algorithm calculated bacterial accumulation (GFP fluorescence) within macrophages, as defined by DAPI and MitoTracker Red. GFP^+^ macrophage area was defined as the number of GFP-positive pixels per macrophage divided by the total number of pixels per macrophage, averaged across all macrophages in the field (13).

### CFU enumeration.

For CFU determination, macrophage infections and treatments were performed as described above, except cells were seeded in standard 96-well tissue culture plates (Greiner catalog number 655185). At 18 h after infection, wells were washed twice with PBS, lysed with 30 μL 0.1% Triton X-100, diluted, plated on agar plates containing STR, and incubated overnight at 37°C. The following day, cell colonies were counted to determine CFU.

### MIC determinations.

To determine bacterial susceptibility to compounds, overnight cultures were grown in MHB, M9, or M9 + EDTA as indicated. Cultures were diluted to a final OD_600_ of 0.01 (~10^7^ cells). Dilutions were distributed in 96-well flat-bottom plates containing test compounds, resulting in the indicated desired final concentration with appropriate vehicle controls. Plates were grown at 37°C with shaking, and the OD_600_ was monitored at 18 h (BioTek Synergy H1 or BioTek Eon). MICs were defined as the concentration at which 99% of growth was inhibited. The clinical MICs were determined starting with 20-fold fewer cells (5 × 10^5^). However, assays with 10^7^ versus 5 × 10^5^ cells yielded similar MICs for JAV1 and JAV 2 (see Fig. S9 in the supplemental material) and had the benefit of reducing variation in the lag periods, facilitating the monitoring of growth over time.

10.1128/mbio.01790-22.9 FIG S9 MIC values for *S.* Typhimurium were similar using initial starting inocula of 5 × 10^5^ and 1 × 10^7^. Comparison of growth in M9 + EDTA (400 μM) in the presence of JAV1 or JAV2 upon inoculation with 1 × 10^7^ (A) or 5 × 10^5^ CFU/mL (B), followed by growth at 37°C with aeration. The clinical standard for MIC assays is to use a starting inoculum of 5 × 10^5^ bacteria, also called a 0.5 McFarland standard (B. Kowalska-Krochmal and R. Dudek-Wicher, Pathogens 10:165, 2021, https://doi.org/10.3390/pathogens10020165). Bacteria grew to a higher OD_600_ by 18 h with the larger inoculum. Download FIG S9, PDF file, 0.2 MB. Copyright © 2022 Villanueva et al. 2022 Villanueva et al. https://creativecommons.org/licenses/by/4.0/ This content is distributed under the terms of the Creative Commons Attribution 4.0 International license.

### Nitrocefin hydrolysis assays.

Nitrocefin hydrolysis assays were performed as described elsewhere (15). Overnight wild-type SL1344 *S.* Typhimurium harboring β-lactamase (*bla*)-expressing pACYC177-mTagBFP2 (110) grown with 30 μg/mL ampicillin were subcultured 1:100, followed by regrowth to mid-log phase (OD_600_ of 0.4 to 0.6). Cells were washed with PBS and resuspended to 10^9^ CFU/mL in the indicated medium conditions. Cells were added to prepared 96-well plates, resulting in tested compounds at the indicated final concentrations along with 100 μM nitrocefin (Sigma catalog number 484400). Absorbance at 486 nm was recorded every minute for 55 min using a BioTek Synergy H1 spectrophotometer. Slopes indicated on graphs were calculated over the entire time course of the experiment.

### Growth curves and kill curves.

Overnight cultures were subcultured 1:100 in M9 + EDTA and incubated at 37°C with aeration until they reached mid-log phase. At time zero, compounds or vehicle control (DMSO) were added to the indicated final concentration. Cultures continued incubating at 37°C with aeration. At the times indicated, aliquots were monitored for OD_600_ and plated for CFU enumeration. Data were normalized to time zero.

### DiSC_3_(5) membrane potential assays.

Membrane potential was measured using the potentiometric fluorescent probe DiSC_3_(5) (Invitrogen catalog number D306). Overnight cultures were subcultured 1:100 in M9 + EDTA and incubated at 37°C with aeration until they reached mid-log phase. Cells were washed with PBS and then diluted to an OD_600_ of 0.4 in M9 + EDTA medium. DiSC_3_(5) was added to a final concentration of 2 μM, and the culture was incubated at 37°C with aeration for 30 min. Cells were distributed into black, 96-well plates (Greiner catalog number 655076). Fluorescence (excitation [ex] at 650 nm and emission [em] at 680 nm) was monitored every 1 min for 20 min of equilibration on a BioTek Synergy H1 plate reader. After baseline fluorescence was recorded, compound was added to the desired final concentration in 1-μL aliquots, cell suspensions were mixed, and measurements were recorded for an additional 30 min. Data were normalized to compound addition (time zero).

### BCECF-AM intracellular pH assays.

Internal pH was measured using the pH-sensitive fluorescent probe BCECF-AM (Molecular Probes catalog number B1170). Overnight cultures were diluted 1:100 in M9 + EDTA and incubated at 37°C with aeration until they reached mid-log phase. BCECF-AM was added to a final concentration of 10 μM and incubated at 37°C with aeration for approximately 1 h. Cells were then distributed into black, 96-well plates (Greiner). Fluorescence (ex 490, em 535 nm and ex 440, em 535 nm) was monitored every 2.5 min during 10 min of equilibration. Compounds were added to the desired final concentration in 1-μL aliquots, cell suspensions were mixed, and fluorescence was monitored every 2.5 min for 30 min using a BioTek Synergy H1 plate reader. BCECF fluorescence was calibrated at seven pH levels between 5.5 and 8 (every 0.5 pH unit). pH was calculated using the equation provided by the manufacturer, with the pK_a_ for BCECF of 6.97 (111). Data were normalized to compound addition (time zero).

### Laurdan membrane fluidity assays.

Membrane fluidity was measured using the lipid density indicator Laurdan (6-dodecanoyl-2-dimethylaminonaphthalene; Invitrogen catalog number D250). These experiments were performed in a 37°C warm room to minimize temperature fluctuations, which alter membrane fluidity (112). Overnight cultures were diluted 1:100 in M9 + EDTA and incubated at 37°C with aeration until they reached mid-log phase. Laurdan was added to a final concentration of 10 μM and incubated at 37°C with aeration for approximately 1 h. Cells were washed using prewarmed PBS and then diluted to an OD_600_ of 0.4 in prewarmed M9 + EDTA medium. Cells were loaded into a prewarmed black, 96-well plate (Greiner), and fluorescence (ex 350, em460 and em 500 nm) was monitored every 2.5 min during 10 min of equilibration. Compounds were added to the desired final concentration in 1-μL aliquots, cell suspensions were mixed, and fluorescence was monitored every 2.5 min for 30 min using a BioTek Synergy H1 plate reader. Data were normalized to compound addition (time zero).

### Propidium iodide membrane integrity assays.

Membrane integrity was monitored using propidium iodide (Life Technologies catalog number P1304MP). This experiment involved frequent removal and transfer of bacterial cultures. To minimize temperature fluctuations, the experiment was conducted in a 37°C warm room. An overnight culture was diluted 1:100 in M9 + EDTA and incubated at 37°C with aeration. At mid-log phase, the culture was split into 11-mL samples, one for each condition. A 0-time point measure was taken to monitor baseline membrane integrity. Then, DMSO, 0.05% SDS, or compound was added to the desired concentration. Five minutes prior to each time point (0 [baseline], 5, 10, 15, 20, and 30 min), 1 mL of sample was harvested, added to a culture tube containing PI at a final concentration of 10 μg/mL, and incubated for 5 min at 37°C with aeration. Cells were pelleted, washed twice with PBS and resuspended in PBS, and monitored for fluorescent signal (ex 535, em 617 nm) using a BioTek Synergy H1 plate reader.

### ATP measurements.

Extra- and intracellular ATP levels were measured using a luminescence-based ATP determination kit (Molecular Probes catalog number A22066) according to the manufacturer’s instructions and as previously described (113). Overnight cultures were diluted 1:100 in M9 + EDTA and incubated at 37°C with aeration until they reached mid-log phase and were then treated as indicated. Aliquots (250 μL) were removed after 30- and 60-min intervals and centrifuged for 5 min. The supernatant from each condition was transferred to a 1.5-mL tube and stored at −80°C. To determine intracellular ATP levels, pelleted bacteria from the extracellular condition were resuspended in 250 μL of culture medium, immediately mixed with ice-cold 1.2 M perchloric acid, and vortexed for 10 s. The mixture was incubated on ice for 15 min and centrifuged at 6,000 × *g* for 5 min at 4°C. Supernatants (200 μL) were transferred to a fresh 1.5-mL tube and mixed with 100 μL of a neutralizing solution containing 0.72 M KOH and 0.16 M KHCO_3_. The neutralized extract was then centrifuged for an additional 5 min, and the supernatant was transferred to a fresh 1.5-mL tube for use for the ATP assay according to the manufacturer’s instructions. Luminescence was recorded on a BioTek Synergy H1 plate reader.

### Liposome sequestration assay.

To assess interaction and sequestration of JAV1 and JAV2 with glycerophospholipids, liposomes were created using the Avanti Mini-Extruder according to the protocol on Avanti’s website (Avanti catalog number 610000; https://avantilipids.com/divisions/equipment-products/mini-extruder-extrusion-technique) and as previously described (114, 115). E. coli total extract (Avanti catalog number 100500C), CL 16:0–18:1 (Avanti catalog number 710341C), and PE 18:0–18:1 (Avanti catalog number 840503C) were diluted to 1 mg/mL in chloroform and mixed by (weight by weight) to the indicated percentages. Lipids were then dried using a Savant SPD1010 SpeedVac Concentrator for 1.5 h. Samples were reconstituted in buffer (100 mM KCl, 25 mM HEPES, 10% glycerol; pH 7.4) to 1 mg/mL for 20 min, and the water bath was sonicated for 7 cycles of sonication (1 min on, 1 min off). The lipid mixture was extruded through 68°C Avanti Mini-Extruder using a 0.1-nm disc filter and accompanying disc filter supports. Lipids were stored at 4°C until use, for a maximum of 2 days. Lipids were equilibrated to room temperature and mixed with 100 μM of either JAV1 or JAV2 and incubated for 10 min. Samples were ultracentrifuged using the Sovall RC M120EX at 100,000 × *g* for 45 min. Supernatants were transferred to a 1.5-mL microcentrifuge tube, and absorbance spectra were quantified using a NanoDrop 2000. *K_d_* values were determined in the same manner using the indicated dilutions of liposomes composed of E. coli extract and 30% CL or PG.

### Resazurin assays.

Resazurin (alamarBlue; Thermo Scientific catalog number AC418900010) was used to assess population reduction potential. Overnight cultures were diluted 1:100 in M9 + EDTA and incubated at 37°C with aeration until they reached mid-log phase. Cells (200 μL per well) were transferred to black, 96-well plates (Greiner) containing compound and resazurin at a final concentration of 100 μg/mL. The plate was incubated with shaking in the dark at room temperature for 5 min. Fluorescence readings were taken every 5 min for 30 min (ex 570, em 650 nm) using a BioTek Synergy H1 plate reader. Data were normalized to the DMSO readings.

### Mitochondrial membrane determination with TMRM.

Experiments were performed with RAW 264.7 cells between passages 1 and 6. Cells were grown in complete DMEM to a confluence of 70 to 90%. Cells were scraped, washed once with PBS, resuspended, and diluted to a final concentration of 5 × 10^5^ cells/mL. Cells (100 μL) were transferred to black, 96-well glass-bottomed plates (Brooks Life Sciences) and incubated for 23.5 h at 37°C with 5% CO_2_. The medium was exchanged for a 100 μL of FluoroBrite DMEM containing 100 nM TMRM. Thirty minutes later, the medium was exchanged for 150 μL of FluoroBrite DMEM. Cells were time-lapse imaged on a Yokogawa CellVoyager CV1000 confocal scanner system with a 40×, 0.60-NA objective and an environmentally controlled chamber for 30 min with images acquired every 10 min prior to compound addition. Compounds were added with a multichannel pipette as 50-μL aliquots to obtain the desired concentration in a final volume of 200 μL. All wells contained a final concentration of 0.4% DMSO except for the CCCP control, which required 0.5% DMSO to remain in solution. Cells were imaged every 10 min for 80 min over 4.5 h and every 30 min for the remainder of the experiment. Two fields of view per well were imaged, with each field comprising five images sampled over a *z*-dimension of 15 μm. Images were converted into maximum intensity projections, and the TMRM foreground signal was extracted via a MATLAB R2018a (MathWorks) script and normalized to time zero for each field.

### LDH assays.

To assess macrophage membrane integrity, the CyQUANT LDH cytotoxicity kit (Invitrogen catalog number C20300) was used according to the manufacturer’s instructions.

### ChemAxon chemical characteristic prediction.

ChemAxon’s Chemicalize platform was used to calculate JAV1 and JAV2 physical chemistry properties, including, lipophilicity c[log(P)], aqueous solubility c[log(S)], and lipophilicity across pH c[log(D) versus pH] values.

### Lysosomal trapping live cell infection microscopy.

RAW 264.7 cells were quantified for LysoTracker Red DND-99 (LTR; Invitrogen L7528) abundance to determine the degree of lysosomal trapping in the presence of indicated compounds (19, 51). Experiments were performed with RAW 264.7 cells between passages 1 and 6. Cells were grown in complete DMEM to a confluence of 70 to 90%. Cells were scraped, washed, resuspended, and diluted to a final concentration of 5 × 10^5^ cells/mL in DMEM. Cells (100 μL) were transferred to black, 96-well glass-bottomed plates (Brooks Life Sciences) and incubated for 24 h at 37°C with 5% CO_2_. Uninfected cells were treated with indicated compounds, DMSO, or 10 mM NH_4_Cl for 1 h. The cell medium was exchanged for 100 μL of complete DMEM containing 50 nM LTR and 9 μM Hoechst 33342 for 30 min. Following staining, the medium was exchanged for 100 μL of FluoroBrite-containing DMEM and imaged on a Yokogawa CellVoyager CV1000 confocal scanner system with a 20×, 0.75-NA objective and an environmentally controlled chamber set to 37°C with 5% CO_2_. Five fields per well were imaged, with each field comprising 5 images sampled over a *z*-dimension of 10 μm. Images were converted into maximum intensity projections, and the integrated LTR signal across macrophage area was quantified using CellProfiler 4.2.1. To monitor LTR during infection, macrophages were seeded as described above and infected with *S.* Typhimurium SL1344 *sifB*::*gfp* as described above for the SAFIRE assay. After 4.5 h, macrophages were treated with compound and imaged as described above. LTR and GFP signals within macrophage area was quantified using CellProfiler 4.2.1.

### Wax moth worm infection.

Galleria mellonella (Carolina catalog number 143928) larvae were sorted and selected based on weight (200 mg and 250 mg), lack of melanization on the thorax and abdomen, and lack of clogged prolegs. G. mellonella larvae were kept at room temperature on shredded woodchips. The volume of G. mellonella was calculated based on an average weight of 225 mg (116). To protect researchers from accidental needle sticks, G. mellonella larvae were restrained using 200-μL tips (117). Antibiotic susceptibility and acute toxicity were undertaken as previously described (118). Infectious and lethal doses were determined to be 1 × 10^7^ and >5 × 10^7^ CFU/mL, respectively. JAV1 and JAV2 were insoluble in aqueous solution at 30 mg/kg, and injecting 10 μL of DMSO into G. mellonella was toxic. Therefore, compounds were formulated in a 1:1:6 ratio of compound:polyethylene glycol BioUltra 300 (Sigma catalog number 90878):water (vol/vol/vol). G. mellonella were infected with approximately 1.7 × 10^5^ CFU/larva in 10 μL in the last left proleg and incubated at 37°C. After 2 h, larvae were injected with 10 μL of compound, CAT, or DMSO formulated as described above in their last right proleg and monitored for mortality every 24 h for 5 days.

### Statistics.

Statistics were preformed using GraphPad Prism version 9.3.1 for Windows (GraphPad Software, San Diego, CA). Comparisons and statistical test information are presented in accordance with the recommendations of Weissgerber et al. (119).
