# Supplementary figures and images for: Salmonella enterica Infections Are Disrupted by Two Small Molecules That Accumulate within Phagosomes and Differentially Damage Bacterial Inner Membranes
Source: mBio. 2022 Sep 22;13(5):e01790-22. doi: 10.1128/mbio.01790-22 (PMC9601186; doi:10.1128/mbio.01790-22)

Figure S1

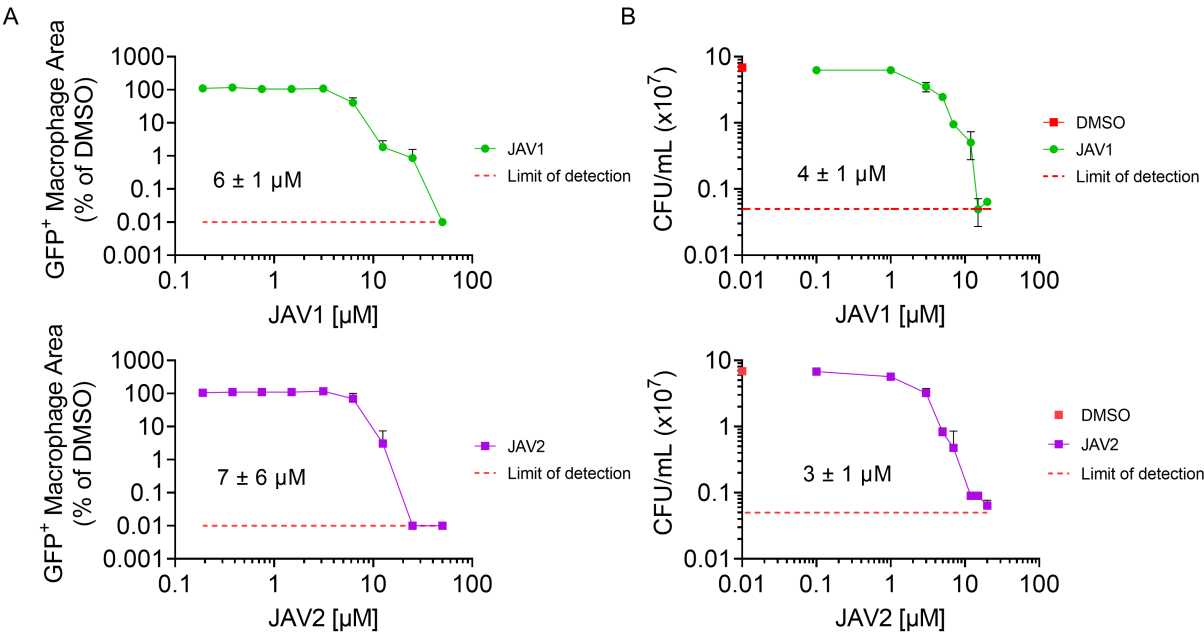

Supplement: FIG S1 [file mbio.01790-22-s0001.pdf]

Figure S2

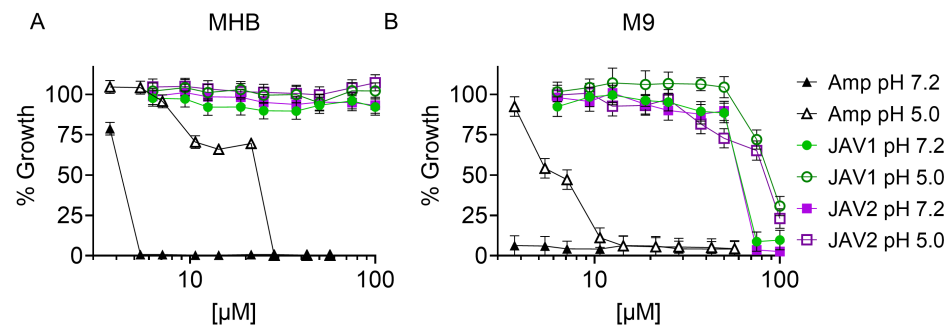

Supplement: FIG S2 [file mbio.01790-22-s0002.pdf]

Figure S3

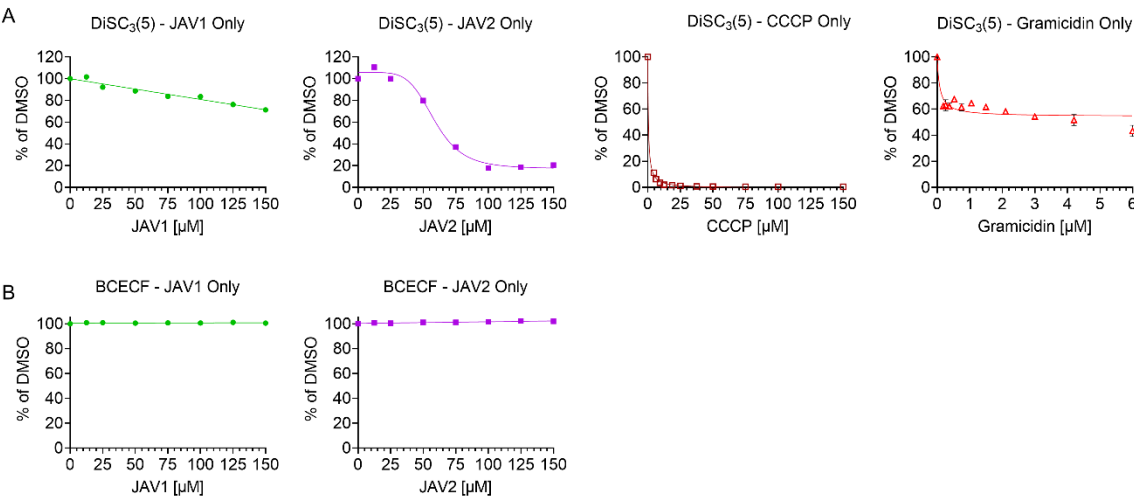

Supplement: FIG S3 [file mbio.01790-22-s0003.pdf]

Figure S5

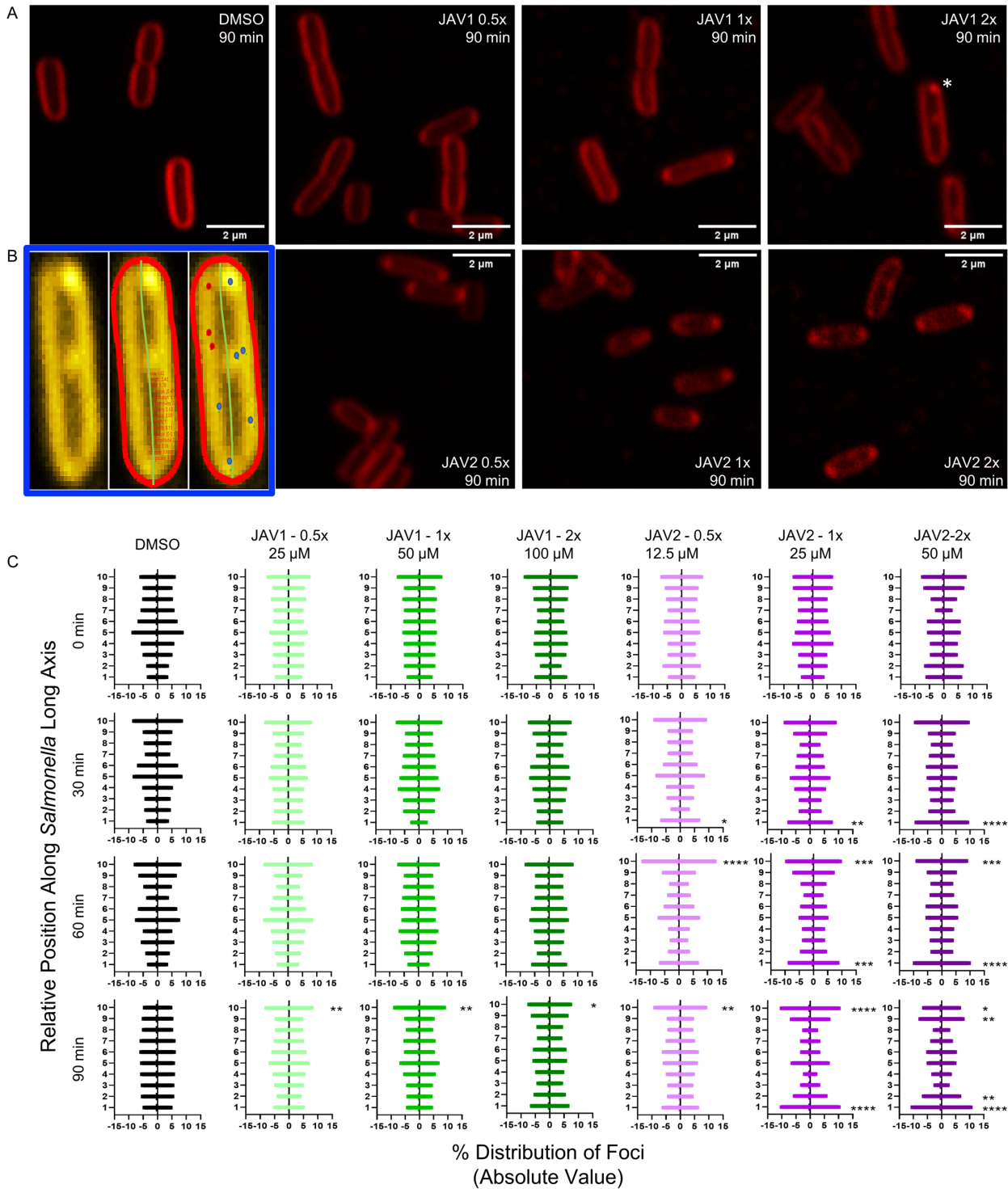

Supplement: FIG S5 [file mbio.01790-22-s0005.pdf]

**Figure S6**

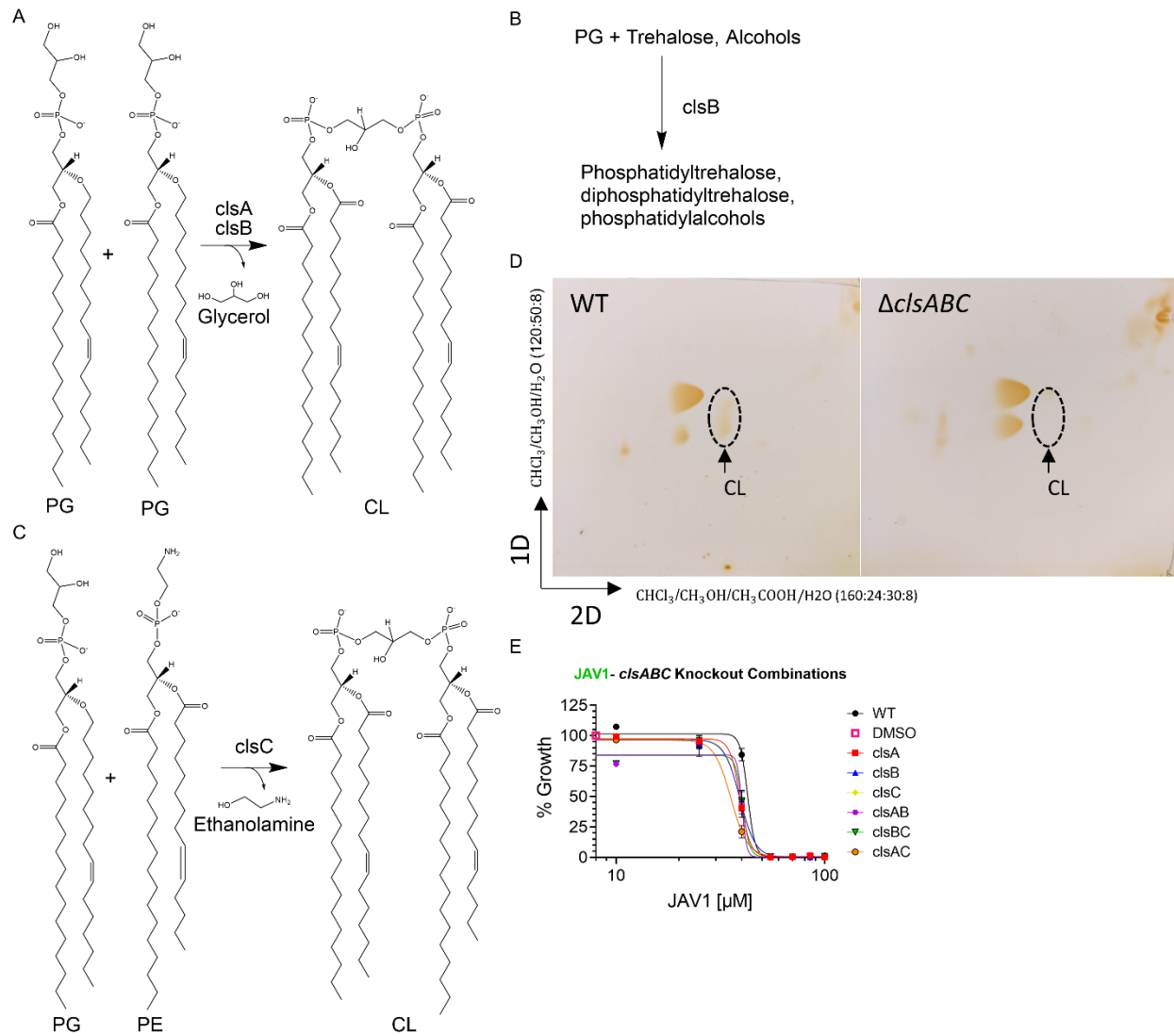

Supplement: FIG S6 [file mbio.01790-22-s0006.pdf]

Figure S7

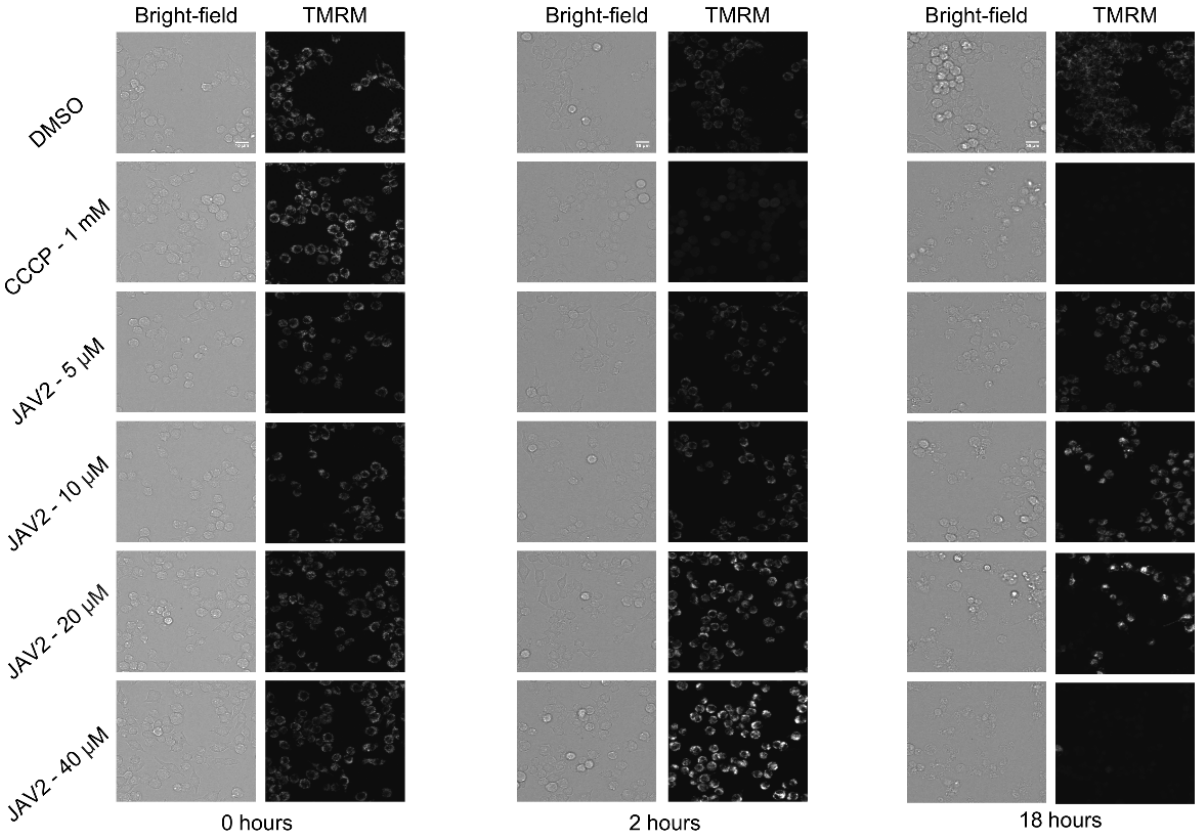

Supplement: FIG S7 [file mbio.01790-22-s0007.pdf]

Figure S8

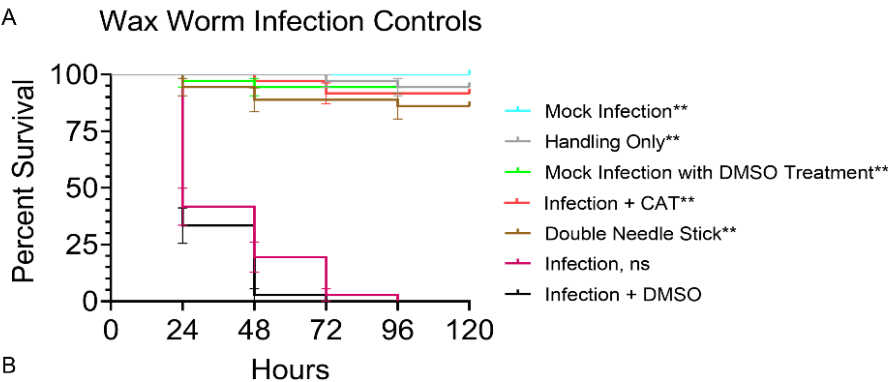

B

| mg/kg | JAV1<br>[μM] | JAV2<br>[μM] | Chloramphenicol<br>(CAT) [μM] |
|-------|--------------|--------------|-------------------------------|
| 30    | 128          | 116          |                               |
| 25    |              |              | 119                           |
| 20    | 85           | 78           |                               |
| 10    | 43           | 39           |                               |
| 5     | 21           | 19           |                               |

Supplement: FIG S8 [file mbio.01790-22-s0008.pdf]

Figure S9

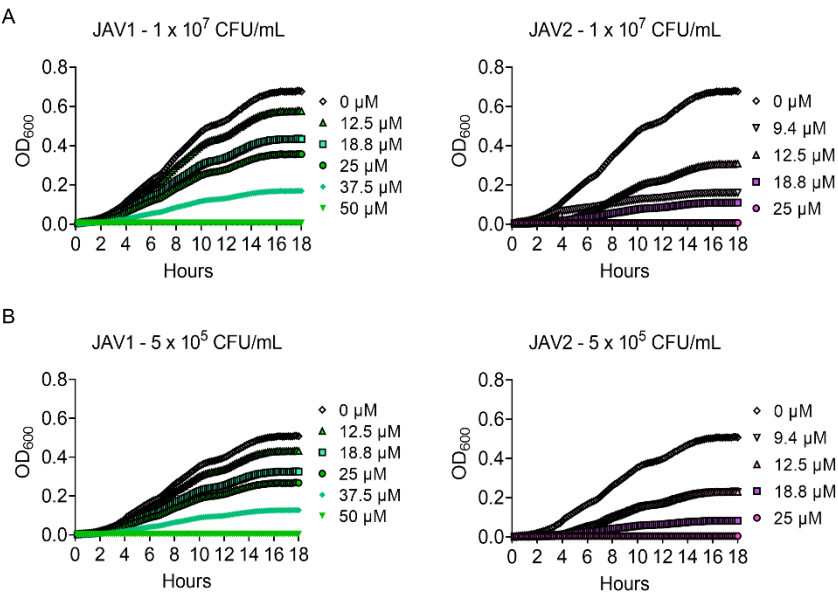

Supplement: FIG S9 [file mbio.01790-22-s0009.pdf]
