# Supplementary material for: Salmonella enterica Infections Are Disrupted by Two Small Molecules That Accumulate within Phagosomes and Differentially Damage Bacterial Inner Membranes
Source: mBio. 2022 Sep 22;13(5):e01790-22. doi: 10.1128/mbio.01790-22 (PMC9601186; doi:10.1128/mbio.01790-22)
Supplement: FIG S4 [file mbio.01790-22-s0004.pdf]

Figure S4

JAV1 / JAV2

A

JAV1

MIC	µM	Phosphatidylethanolamine										
		0	0.4	0.7	1.5	3.7	7.5	18.7	37.3	74.7	112.0	µg/mL
MIC	µM	0	0.5	1	2	5	10	25	50	100	150	µM
4x	200											
3x	150											
2x	100											
1.5x	75											
1x	50											
DMSO	DMSO											

Charge	Log P (Lipophilicity)
Zwitterion	9.2

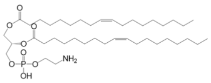

JAV2

MIC	µM	Phosphatidylethanolamine										
		0	0.4	0.7	1.5	3.7	7.5	18.7	37.3	74.7	112.0	µg/mL
MIC	µM	0	0.5	1	2	5	10	25	50	100	150	µM
4x	150											
3x	100											
2x	50											
1.5x	37.5											
1x	25											
DMSO	DMSO											

Scale
150%
100%
80%
60%
50%
40%
20%
0%

B

JAV1

MIC	µM	Phosphatidylglycerol										
		0	0.2	0.5	1.0	2.5	5.0	12.4	24.8	49.6	74.3	µg/mL
MIC	µM	0	0.5	1	2	5	10	25	50	100	150	µM
4x	200											
3x	150											
2x	100											
1.5x	75											
1x	50											
DMSO	DMSO											

Charge	Log P (Lipophilicity)
-1	9.7

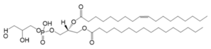

JAV2

MIC	µM	Phosphatidylglycerol										
		0	0.2	0.5	1.0	2.5	5.0	12.4	24.8	49.6	74.3	µg/mL
MIC	µM	0	0.5	1	2	5	10	25	50	100	150	µM
4x	150											
3x	100											
2x	50											
1.5x	37.5											
1x	25											
DMSO	DMSO											

C

JAV1

MIC	µM	Kdo$_2$-Lipid A										
		0	1.2	2.3	4.6	11.5	23.1	57.7	115.3	231.0	346.0	µg/mL
MIC	µM	0	0.5	1	2	5	10	25	50	100	150	µM
4x	200											
3x	150											
2x	100											
1.5x	75											
1x	50											
DMSO	DMSO											

Charge	Log P (Lipophilicity)
-2	10.7

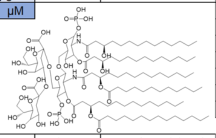

JAV2

MIC	µM	Kdo$_2$-Lipid A										
		0	1.2	2.3	4.6	11.5	23.1	57.7	115.3	231.0	346.0	µg/mL
MIC	µM	0	0.5	1	2	5	10	25	50	100	150	µM
4x	150											
3x	100											
2x	50											
1.5x	37.5											
1x	25											
DMSO	DMSO											

D

JAV1

MIC	µM	Cardiolipin										
		0	0.7	1.4	2.9	7.2	14.5	36.2	72.5	145.0	217.5	µg/mL
MIC	µM	0	0.5	1	2	5	10	25	50	100	150	µM
4x	200											
3x	150											
2x	100											
1.5x	75											
1x	50											
DMSO	DMSO											

Charge	Log P (Lipophilicity)
-2	10.7

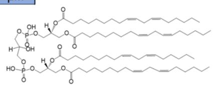

JAV2

MIC	µM	Cardiolipin										
		0.0	0.7	1.4	2.9	7.2	14.5	36.2	72.5	145.0	217.5	µg/mL
MIC	µM	0	0.5	1	2	5	10	25	50	100	150	µM
4x	150											
3x	100											
2x	50											
1.5x	37.5											
1x	25											
DMSO	DMSO											
